# Evaluating the therapeutic potential of L-arginine and licorice extract in letrozole-induced polycystic ovary syndrome in rats: targeting interconnected molecular pathways

**DOI:** 10.1038/s41598-026-63294-5

**Published:** 2026-08-24

**Authors:** Yasmeen El-Sayed, Heba A. El-Ghaweet, Abdelaziz A. Elrefaeey, Amoura M. Abou-El-Naga

**Affiliations:** 1 Faculty of Science, Zoology Department, Mansoura University, Mansoura, Egypt; 2 Faculty of Medicine, Obstetrics and Gynecology Department, Mansoura University, Mansoura, Egypt; 3 Assistant Lecturer of Embryology, Faculty of Science, Zoology Department, Mansoura University, Mansoura, Egypt

**Keywords:** Polycystic ovarian syndrome (PCOS), L-arginine, Licorice, Antioxidants, Amhr2 gene, And Rats, Biochemistry, Zoology, Diseases, Medical research

## Abstract

Polycystic Ovary Syndrome (PCOS) is a common disorder characterized by hyperandrogenism and ovarian dysfunction. L-arginine and licorice extract, known for their antioxidant properties, are used to manage infertility. This study aimed to evaluate the effects of licorice extract and L-arginine on ovarian morphology, oocyte maturation, and pregnancy rates in PCOS-induced rats. Female rats were randomly assigned into six groups (n = 12): Group I (Control) received no treatment; Group II (Licorice) received 150 mg/kg licorice extract for 21 days; Group III (L-Arginine) received 22.9 mg/kg L-arginine for 21 days; Group IV (PCOS Model) received 1 mg/kg letrozole for 21 days to induce PCOS; Group V (Licorice + PCOS) received 150 mg/kg licorice extract post-PCOS induction; Group VI (L-Arginine + PCOS) received 22.9 mg/kg L-arginine post-PCOS induction. The study analyzed ovarian morphology, serum lipid profiles, antioxidant markers, nitric oxide levels, inflammatory cytokines, reproductive hormones, and Amhr2 gene expression, in conjunction with ovarian histopathology and immunohistochemistry for PCNA and Ki-67. L-arginine and licorice extract significantly improved lipid profiles, reduced oxidative stress (MDA, NO), and enhanced antioxidant activity (SOD, Catalase). They also showed beneficial effects on ovarian function, inflammation, and reproductive hormones, suggesting their therapeutic potential in managing PCOS. Fertility success improved from 33.3% in PCOS rats to 100% in both treatment groups, with increased litter sizes (9.25 ± 0.63 and 8.25 ± 0.75, respectively).

## Introduction

Polycystic Ovary Syndrome (PCOS) is a common endocrine disorder in up to 10% of women of reproductive age globally. It is characterized by polycystic ovaries, hyperandrogenism, and ovulatory dysfunction, causing menstrual irregularities, infertility, and hirsutism. Beyond reproduction, PCOS is linked to metabolic issues like insulin resistance(IR), dyslipidemia, and central obesity, increasing the risk of type 2 diabetes and cardiovascular diseases^1^. PCOS is a heterogeneous functional endocrine disorder that is linked to low-grade chronic inflammation^2^. PCOS is believed to be the most common cause of anovulatory infertility in women. PCOS has a complex, multifactorial pathogenesis. Growing evidence has pointed to the critical involvement of abnormal energy metabolism and oxidative stress in granulosa cells (GCs) in abnormal follicle development and decreased fertility in PCOS patients. Women with PCOS frequently have mitochondrial dysfunction, higher oxidative stress, and aberrant glucose metabolism, all of which can negatively affect oocyte quality^3^.

Currently, available treatment modalities for PCOS include lifestyle modifications, insulin-sensitizers (Metformin), hormonal contraceptives, anti-androgens, ovulation induction agents, and assisted reproductive techniques (ARTs) in cases of infertility. The treatment choice is usually individualised according to the predominant clinical manifestations and reproductive goals of the patient. Although there are established clinical benefits, responses to treatment may differ between patients, and the choice of therapy often requires consideration of individual efficacy, safety, and tolerability, as well as reproductive goals. Thus, the search for additional therapeutic strategies targeting the metabolic, inflammatory, oxidative, and reproductive disturbances related to PCOS is an ongoing field of research^4^.

Licorice (*Glycyrrhiza glabra*), used in traditional medicine, contains glycyrrhizin and flavonoids with anti-inflammatory, antioxidant, and endocrine-modulating effects^5^. It may reduce androgens, improve insulin sensitivity, and mitigate oxidative stress^6^. The positive benefits of the licorice are assigned to its major ingredients, such as the glycyrrhizinic acid, glabridin, and iso-liquiritigenin. However, the effects of licorice supplementation on lipid profiles were inconsistent. Therefore, some studies have shown beneficial effects of licorice supplementation on total cholesterol (TC) and low-density lipoprotein cholesterol, whereas others did not find any effects. Moreover, several clinical studies reported significant improvements in IR and fasting insulin concentrations after supplementation with licorice and Glabridin, a major component of licorice^7^, making it a promising natural treatment for PCOS.

Another promising therapeutic agent is L-arginine. L-arginine is a precursor of nitric oxide, which improves vascular function, insulin signalling, and reduces oxidative stress. In addition to its metabolic benefits, nitric oxide plays a significant role in ovarian angiogenesis, follicular development, and oocyte quality, indicating a potential therapeutic role for L-arginine in enhancing reproductive outcomes in PCOS^8,9^.

This study evaluates the effects of licorice extract or L-arginine on metabolic parameters, oxidative stress markers, hormonal profiles, and ovarian histology in a letrozole-induced PCOS animal model. The findings could support these agents as safer, holistic alternatives to conventional therapies for PCOS, while minimizing the side effects of the conventional therapies.

## Results

GC/MS Profiling of Licorice Ethanol Extracts: The GC–MS analysis of licorice ethanol extract identified a range of volatile constituents, including flavonoids, saponins, and phytoestrogens, summarized in Table 1. These compounds were classified into 25 chemical categories, with notable constituents such as 4’-O-Methylglabridin, Glabridin, Xyshalogenin, 20-(3-butynyl)-Pregna-5,7-dien-3-ol, DL-Tyrosine, 4-Vinylguaiacol, and Phloretic acid, which exhibited significant peak areas (%).

“Only major compounds identified by GC-MS are shown. For clarity, compounds with low relative abundances are not presented. The sum of relative abundances of listed compounds is 84.70% instead of 100%.”

**Table.1:** Chemical constituents identified by the GC/MS technique of Licorice Ethanol Extracts.

Chemical name	Retention Time (RT, min.)	Molecular Weight	Molecular Formula	Composition% (Area %)
Diglycerol	5.81	166	C_6_H_14_O_5_	1.22
3,5-Dihydroxy-6-methyl-2,3-dihydro-4H-pyran-4-one	8.19	144	C_6_H_8_O_4_	1.04
2-Piperidinylacetic acid	9.45	143	C_7_H_13_NO_2_	1.17
4-Vinylguaiacol	12.38	150	C_9_H_10_O_2_	1.77
2,3,4,5-Tetrahydroxypentanal	17.02	150	C_5_H_10_O_5_	0.81
3,3,7-Trimethyl-2-benzofuran-1(3H)-one	17.38	176	C_11_H_12_O_2_	3.05
3-Hydroxy-4,6-di-T-butyl-2H-Pyran-2-One	18.34	224	C_13_H_20_O_3_	0.56
Phloretic acid	21.89	166	C_9_H_10_O_3_	0.39
DL-Tyrosine	22.03	181	C_9_H_11_NO_3_	0.39
Mome Inositol	23.93	194	C_7_H_14_O_6_	4.36
1,2-Benzenedicarboxylic acid, bis (2-methylpropyl) ester	24.71	278	C_16_H_22_O_4_	0.85
Palmitic acid, methyl ester	26.43	270	C_17_H_34_O_2_	1.06
n-Hexadecanoic acid	27.33	256	C_16_H_32_O_2_	10.18
methyl heptadecanoate	27.76	284	C_18_H_36_O_2_	0.27
8,11-Octadecadienoic acid, methyl ester	29.46	294	C_19_H_34_O_2_	1.69
Methyl 11-octadecenoate	29.64	296	C_19_H_36_O_2_	1.31
cis-Linoleic acid	30.37	280	C_18_H_32_O_2_	13.98
Oleic acid	30.51	282	C_18_H_34_O_2_	14.41
Stearic acid	30.96	284	C_18_H_36_O_2_	2.59
1,3-dihydroxypropan-2-yl (E)-octadec-9-enoate	38.86	356	C_21_H_40_O_4_	0.73
20-(3-butynyl)-Pregna-5,7-dien-3-ol	40.04	352	C_25_H_36_O	1.24
Xyshalogenin	40.17	372	C_23_H_32_O_4_	1.12
Di(pentamethylphenyl)ketone	41.40	322	C_23_H_30_O	2.53
4'-O-Methylglabridin	42.28	338	C_21_H_22_O_4_	6.78
Glabridin	43.78	324	C_20_H_20_O_4_	10.59
				**84.70**

**Fig.1:**
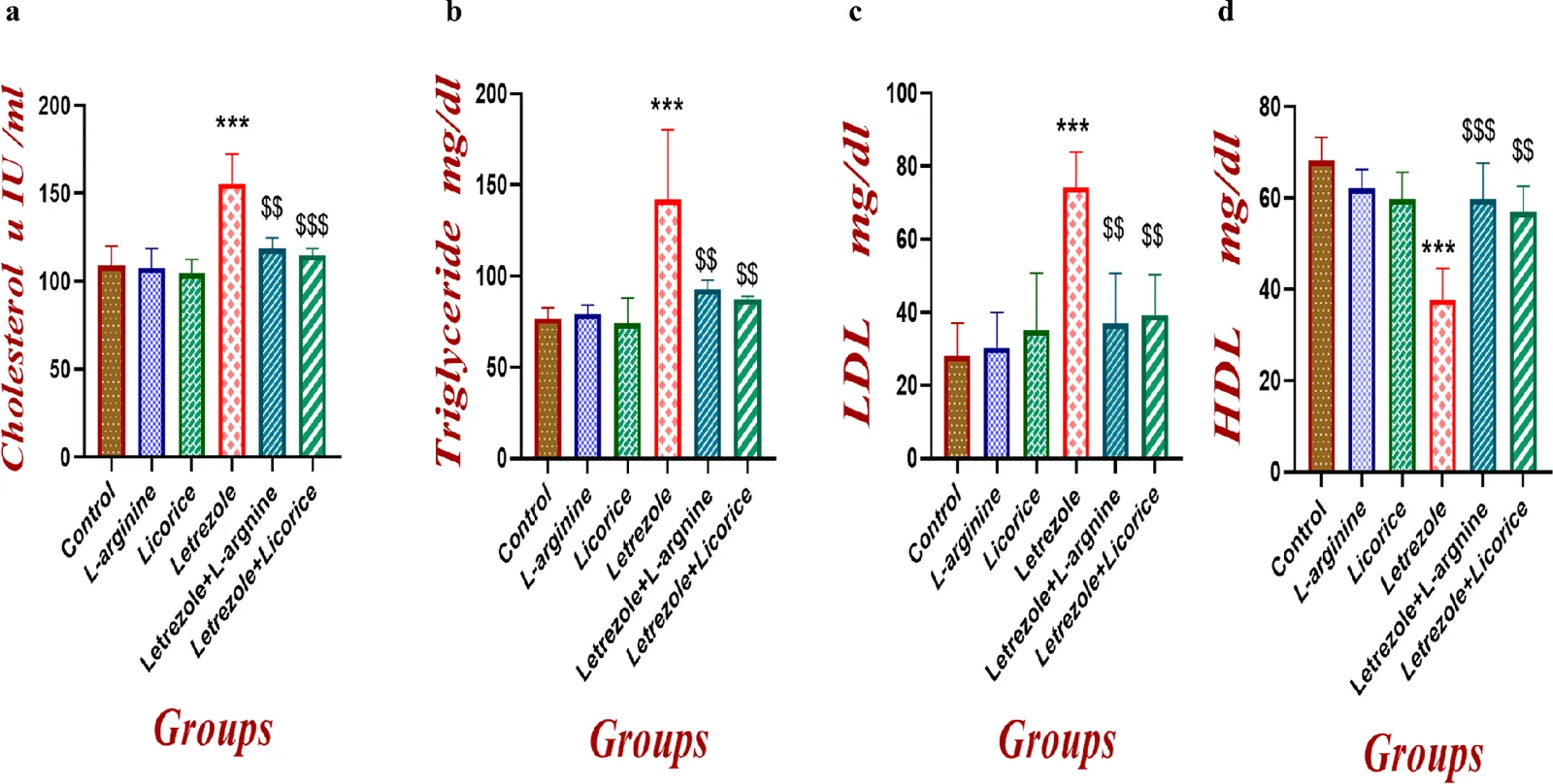
Effect of Letrozole, L-arginine & Licorice on Lipid Profile. (u IU /ml; **a**), Cholesterol, (mg/dl; **b**), Triglyceride, (mg/dl; c), LDL, & (mg/dl; d) HDL in different rat groups. Data are shown as mean ± SEM (*n* = 6)*.* ^**$$**^ Significant at *P* < *0.01* and ^***, $$$^ Significant at *P* < *0.001.* ^***^ Indicate comparisons with respect to the control group. ^**$**^ Indicate comparisons with respect to the PCOS group. *Cont*: control, *Letrozole:* PCOS, *Letrozole* + *L-arginine:* PCOS + L-arginine, *Letrozole* + *Licorice:* PCOS + Licorice.

The lipid profile of PCOS-induced female rats showed a significant increase in serum total cholesterol, triglycerides, and LDL levels (*P* ≤ 0.001), accompanied by a substantial decrease in HDL levels (*P* ≤ 0.001) compared to the control group (Fig. 1a–d). Treatment with L-arginine or licorice extract significantly reduced serum cholesterol, triglycerides, and LDL levels, increasing HDL levels, and bringing them closer to control group values. These results suggest that L-arginine or licorice extract helps normalize lipid profiles in PCOS-induced rats.

**Fig.2:**
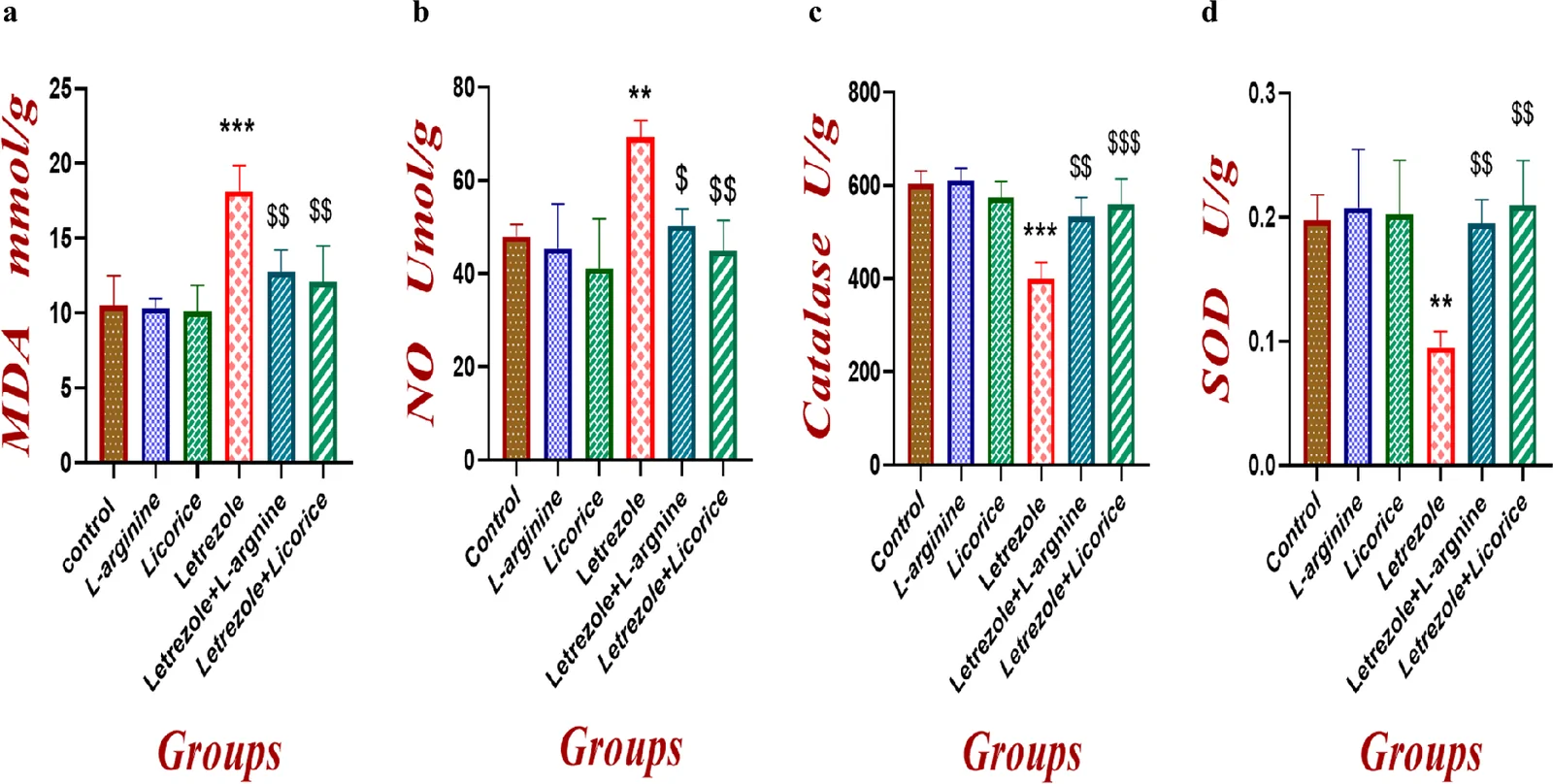
Effect of Letrozole, L-arginine & Licorice on Antioxidants and Oxidative Stress. (mmol/g; **a**), MDA, (Umol/g; **b**), NO, (U/g; c), Catalase & (U/g; d) SOD in different rat groups. Data are shown as mean ± SEM (*n* = 6). ^$^ Significant at *P* < *0.05*, ^**, $$^ Significant at *P* < *0.01* and ^***, $$$^ Significant at *P* < *0.001.* ^**, ***^ Indicate comparisons with respect to the control group. ^$, $^ Indicate comparisons with respect to the PCOS group. *Cont*: control, *Letrozole:* PCOS, *Letrozole* + *L-arginine:* PCOS + L-arginine, *Letrozole* + *Licorice:* PCOS + Licorice.

Oxidative stress and antioxidant activity were also assessed in the ovarian tissues. Polycystic rats significantly increased MDA and NO levels (*P* ≤ 0.001 and *P* ≤ 0.01, respectively) while reducing catalase and SOD activity (*P* ≤ 0.001 and *P* ≤ 0.01, respectively) in PCOS rats compared to controls (Fig. 2a–d). In contrast, L-arginine or licorice extract treatment significantly reduced MDA and NO levels, while increasing catalase and SOD activity in the ovaries compared to untreated PCOS rats. These findings suggest that L-arginine and licorice extract have antioxidant effects, which may help alleviate oxidative stress associated with PCOS.

**Fig. 3:**
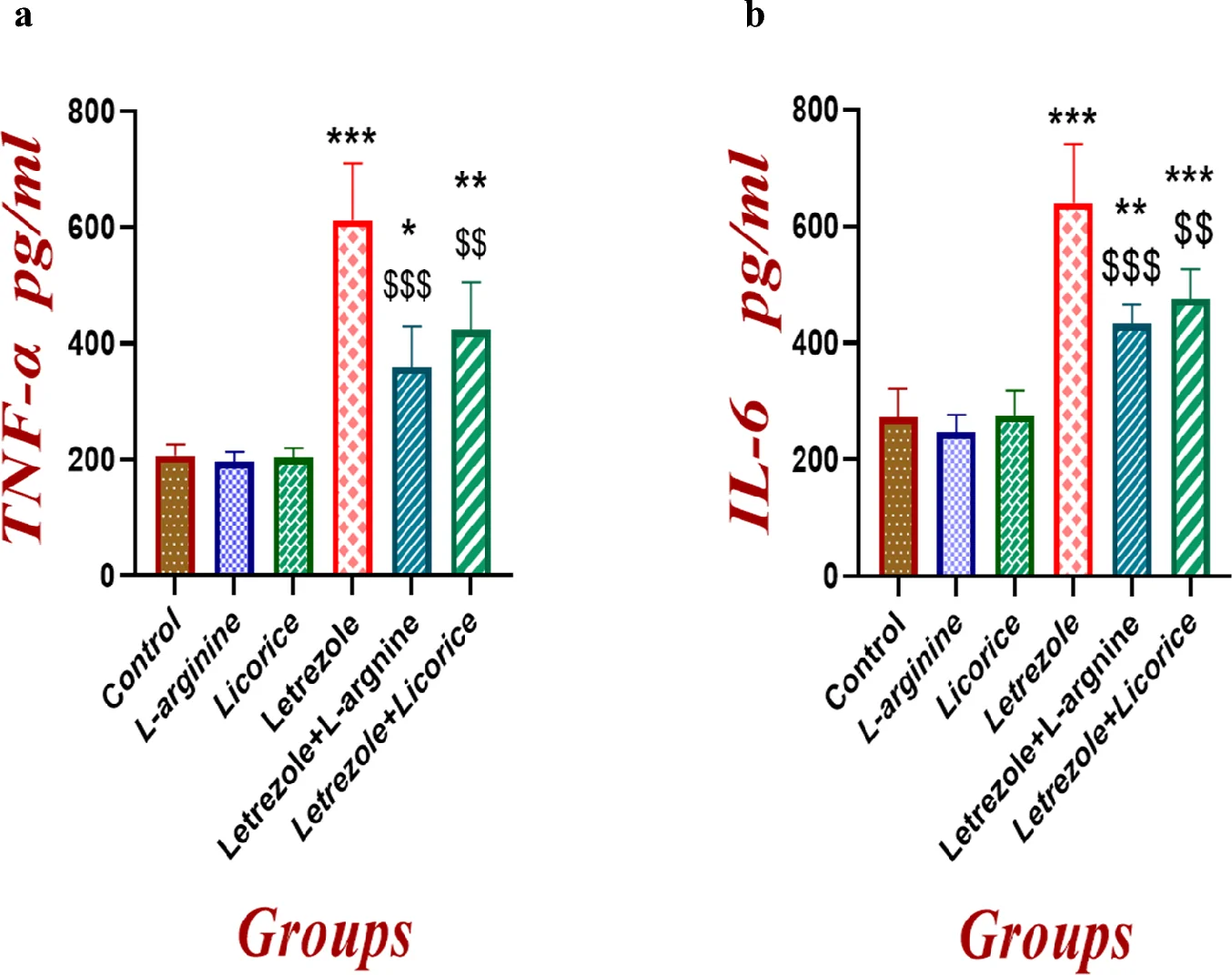
Effect of Letrozole, L-arginine & Licorice on Pro-inflammatory cytokines. (Pg/ml; **a**), TNF-α & (Pg/ml; **b**), IL-6 in different rat groups. Data are shown as mean ± SEM (*n* = 6). ^*^ Significant at *P* < *0.05*, ^**, $$^ Significant at *P* < *0.01* and ^***, $$$^ Significant at *P* < *0.001.* ^*, **, ***^ Indicate comparisons with respect to the control group. ^$^ Indicate comparisons with respect to the PCOS group. *Cont*: control, *Letrozole:* PCOS, *Letrozole* + *L-arginine:* PCOS + L-arginine, *Letrozole* + *Licorice:* PCOS + Licorice.

Rats with PCOS revealed elevated serum levels of pro-inflammatory markers, TNF-α, and IL-6 (*P* ≤ 0.001 and *P* ≤ 0.01, respectively), indicating a chronic low-grade inflammatory state (Fig. 3a–b). However, L-arginine or licorice extract treatment significantly reduced these cytokine levels in PCOS rats (Fig. 3a–b).

**Fig.4:**
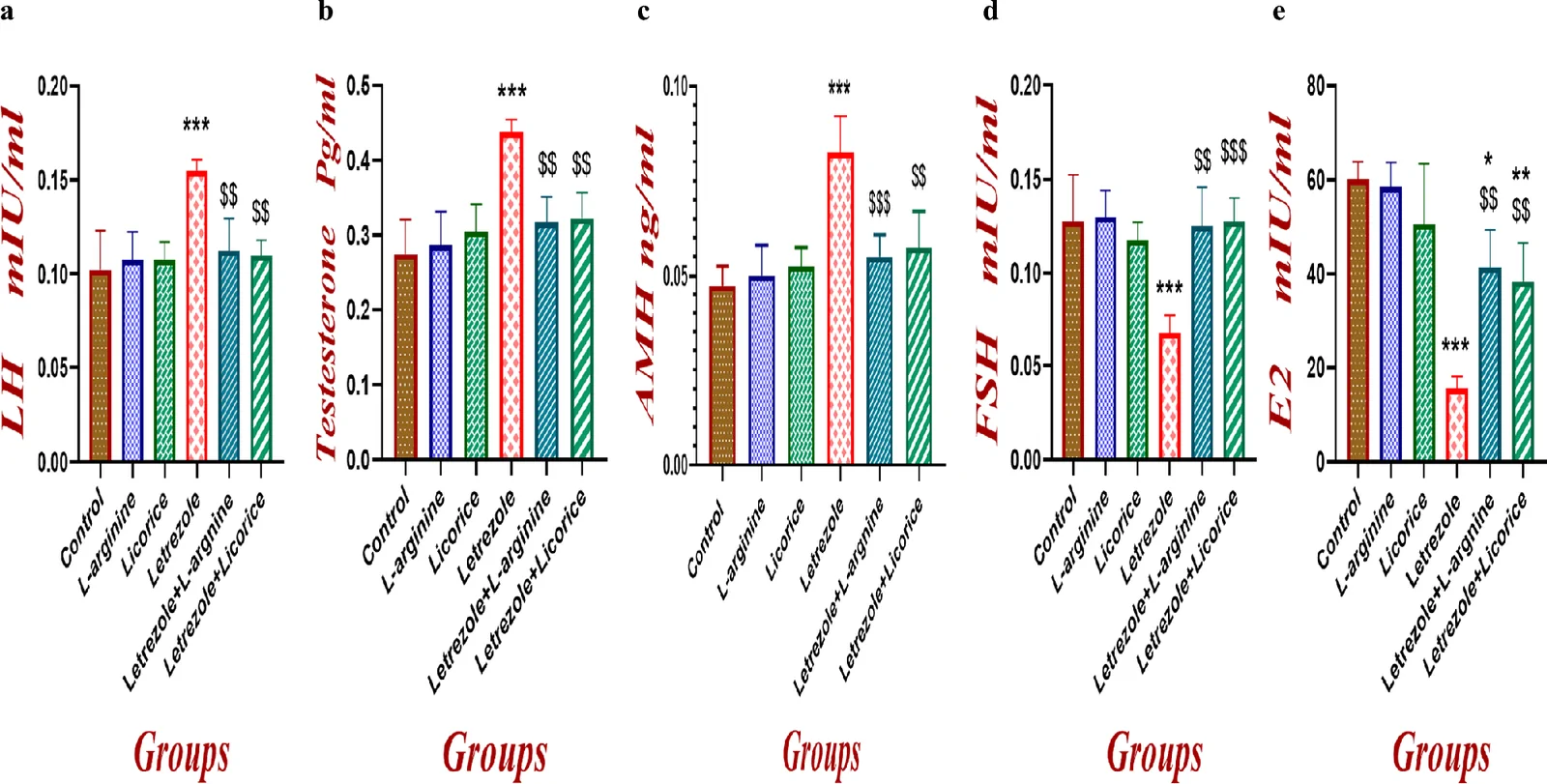
Effect of Letrozole, L-arginine & Licorice on hormones. (mIU/ml; **a**), LH, (Pg/ml; **b**), T, (ng/ml; **c**), AMH, (mIU/ml; **d**), FSH & (mIU/ml; **e**) E2 in different rat groups. Data are shown as mean ± SEM (*n* = 6). ^*^ Significant at *P* < *0.05*, ^**, $$^ Significant at *P* < *0.01* and ^***, $$$^ Significant at *P* < *0.001.* ^*, **, ***^ Indicate comparisons with respect to the control group. ^**$**^ Indicate comparisons with respect to the PCOS group.*Cont*: control, *Letrozole:* PCOS, *Letrozole* + *L-arginine:* PCOS + L-arginine, *Letrozole* + *Licorice:* PCOS + Licorice.

Letrozole-treated female rats resulted in increased levels of LH, testosterone, and AMH (*P* ≤ 0.001), while FSH and E2 levels decreased (*P* ≤ 0.001) compared to controls (Fig. 4a–e). L-arginine or licorice extract treatment significantly lowered LH, testosterone, and AMH levels, while increasing FSH and E2 levels in PCOS rats (Fig. 4a–e).

**Fig.5:**
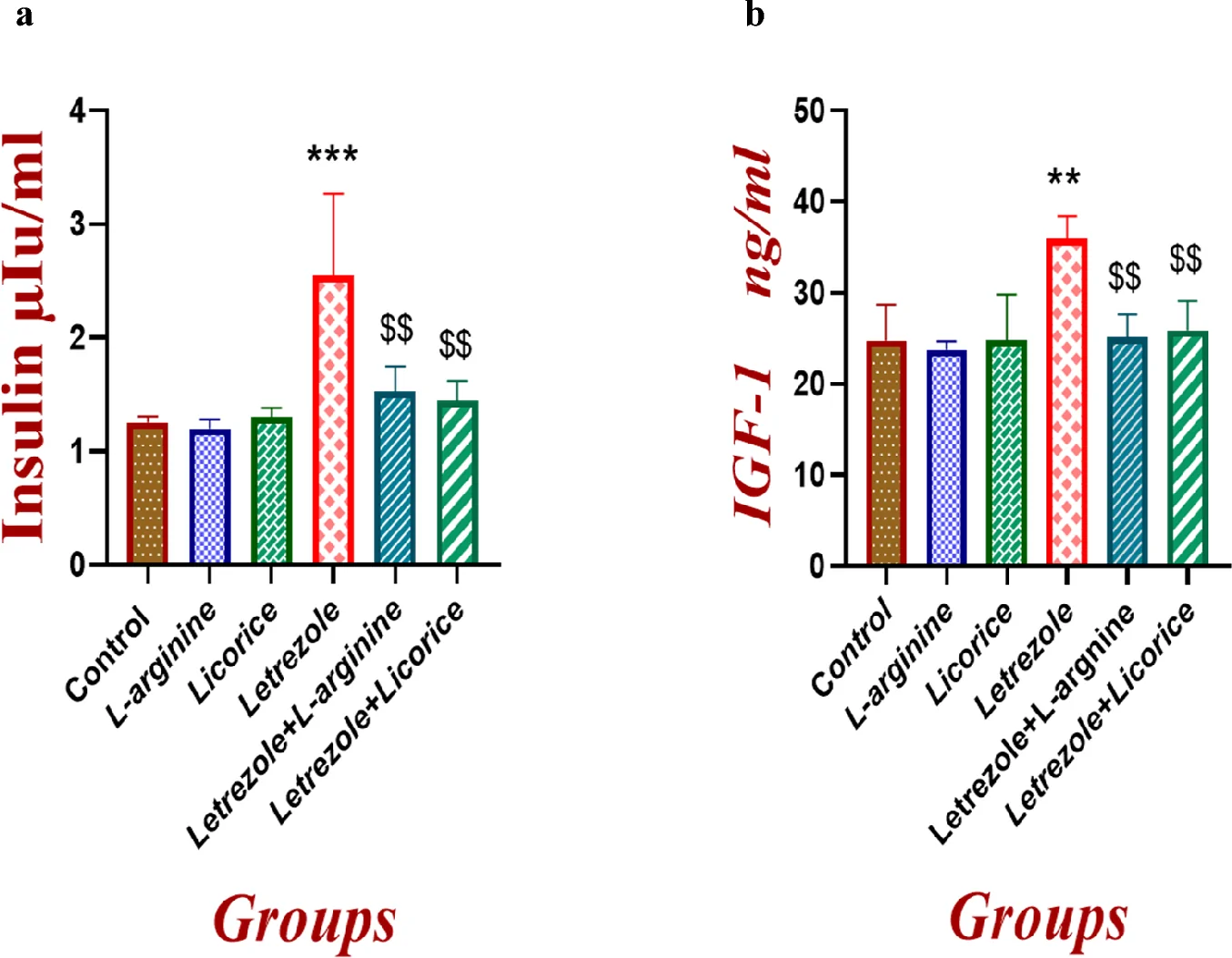
Effect of Letrozole, L-arginine & Licorice on (µIu/ml; a), insulin & (ng/ml; b), IGF-1 in different rat groups. Data are shown as mean ± SEM (*n* = 6). ^**, $$^ Significant at *P* < *0.01* and ^***^ Significant at *P* < *0.001.* ^**, ***^ Indicate comparisons with respect to the control group. ^**$$**^ Indicate comparisons with respect to the PCOS group. *Cont*: control, *Letrozole:* PCOS, *Letrozole* + *L-arginine:* PCOS + L-arginine, *Letrozole* + *Licorice:* PCOS + Licorice.

PCOS rats exhibited significantly higher insulin hormone and IGF-1 levels (*P* ≤ 0.001 and *P* ≤ 0.01, respectively), which were reduced following L-arginine or licorice extract treatment (Fig. 5a–b).

**Fig.6:**
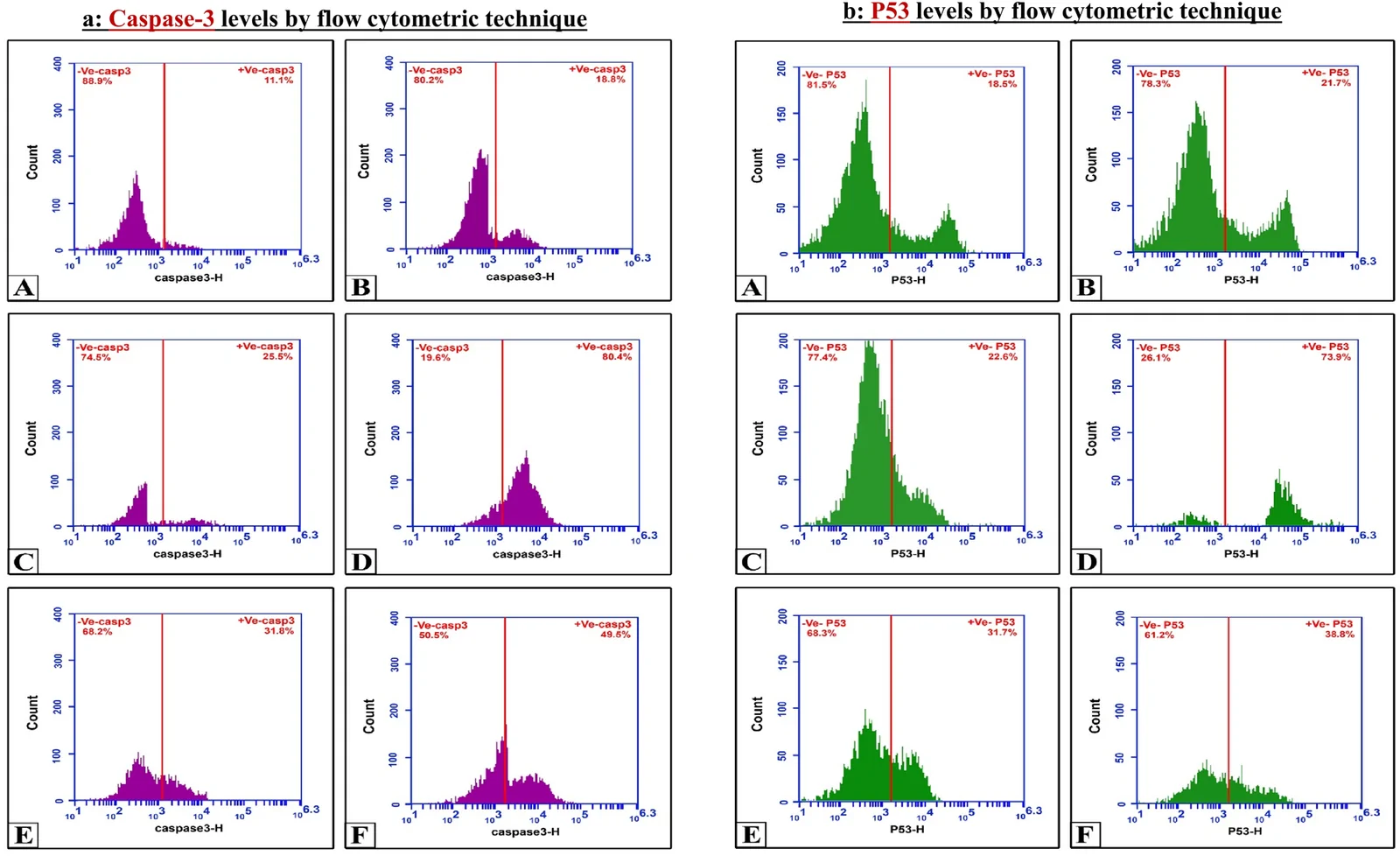
Shows representative flow cytometry histograms. (%; a), Caspase-3 levels by flow cytometric technique in different rat groups; **A** control-casp3, **B** L-arginine-casp3, **C** Licorice-casp3, **D** Letrozole (PCOS) -casp3, **E** Letrozole + L-arginine-casp3, **F** Letrozole + Licorice-casp3 & (%; b), P53 levels by flow cytometric technique in different rat groups; **A** control- P53, **B** L-arginine- P53, **C** Licorice- P53, **D** Letrozole (PCOS)—P53, **E** Letrozole + L-arginine- P53, **F** Letrozole + Licorice- P53.

**Fig.7:**
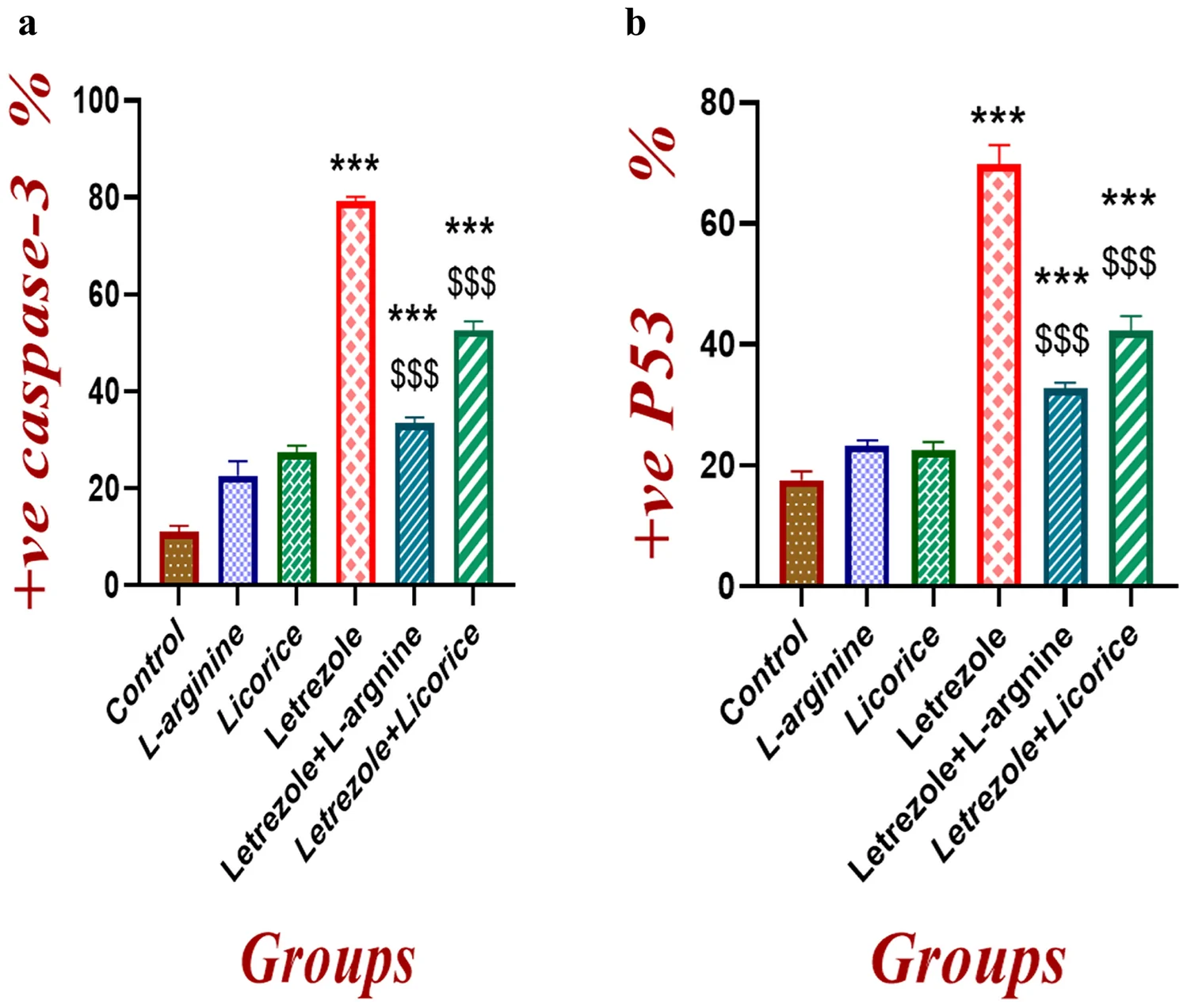
Shows the quantified percentage of positive cells. (%; **a**), Caspase-3 & (%; **b**), P53 in different rat groups. Data are shown as mean ± SEM (*n* = 6). ^***, $$$^ Significant at *P* < *0.001.* ^***^ Indicate comparisons with respect to the control group. ^**$$$**^ Indicate comparisons with respect to the PCOS group. *Cont*: control, *Letrozole:* PCOS, *Letrozole* + *L-arginine:* PCOS + L-arginine, *Letrozole* + *Licorice:* PCOS + Licorice.

Caspase-3 and P53 expression, markers of apoptosis, were significantly higher in the ovaries of PCOS rats (*P* ≤ 0.001) (Figs. 6a–b, 7a-b). However, treatment with L-arginine or licorice extract significantly reduced Caspase-3 and P53 expression in these rats (Fig. 6a-b, 7a-b).

**Fig.8:**
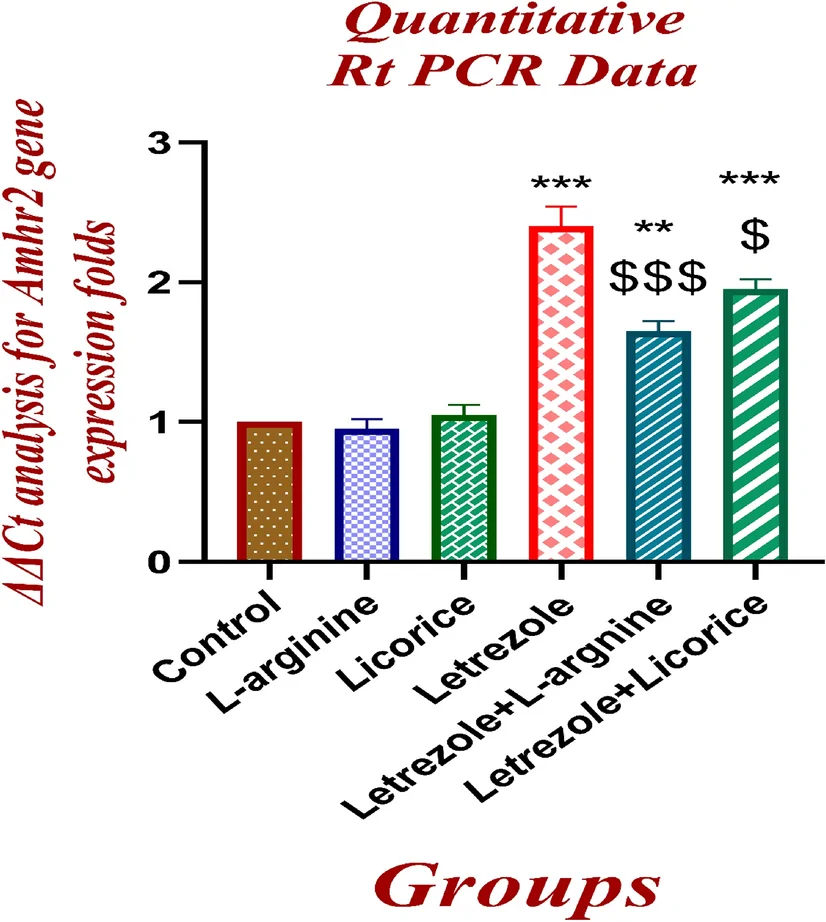
Effect of Letrozole, L-arginine & Licorice on quantitative RT PCR Data. Amhr2 gene expression in different rat groups. Data are shown as mean ± SEM (*n* = 6).^**$**^ Significant at *P* < *0.05*, ^**^ Significant at *P* < *0.01* and ^***, $$$^ Significant at *P* < *0.001.* ^**, ***^ Indicate comparisons with respect to the control group. ^**$, $$$**^ Indicate comparisons with respect to the PCOS group. *Cont*: control, *Letrozole:* PCOS, *Letrozole* + *L-arginine:* PCOS + L-arginine, *Letrozole* + *Licorice:* PCOS + Licorice.

Amhr2 gene expression levels in the ovarian tissue were significantly higher in PCOS-induced rats compared to the control group (*P* ≤ 0.001) (Fig. 8). However, qPCR analysis revealed a significant decrease in Amhr2 gene expression following the administration of L-arginine or licorice extract to PCOS-induced female rats compared to the untreated PCOS group (Fig. 8).

**Fig. 9:**
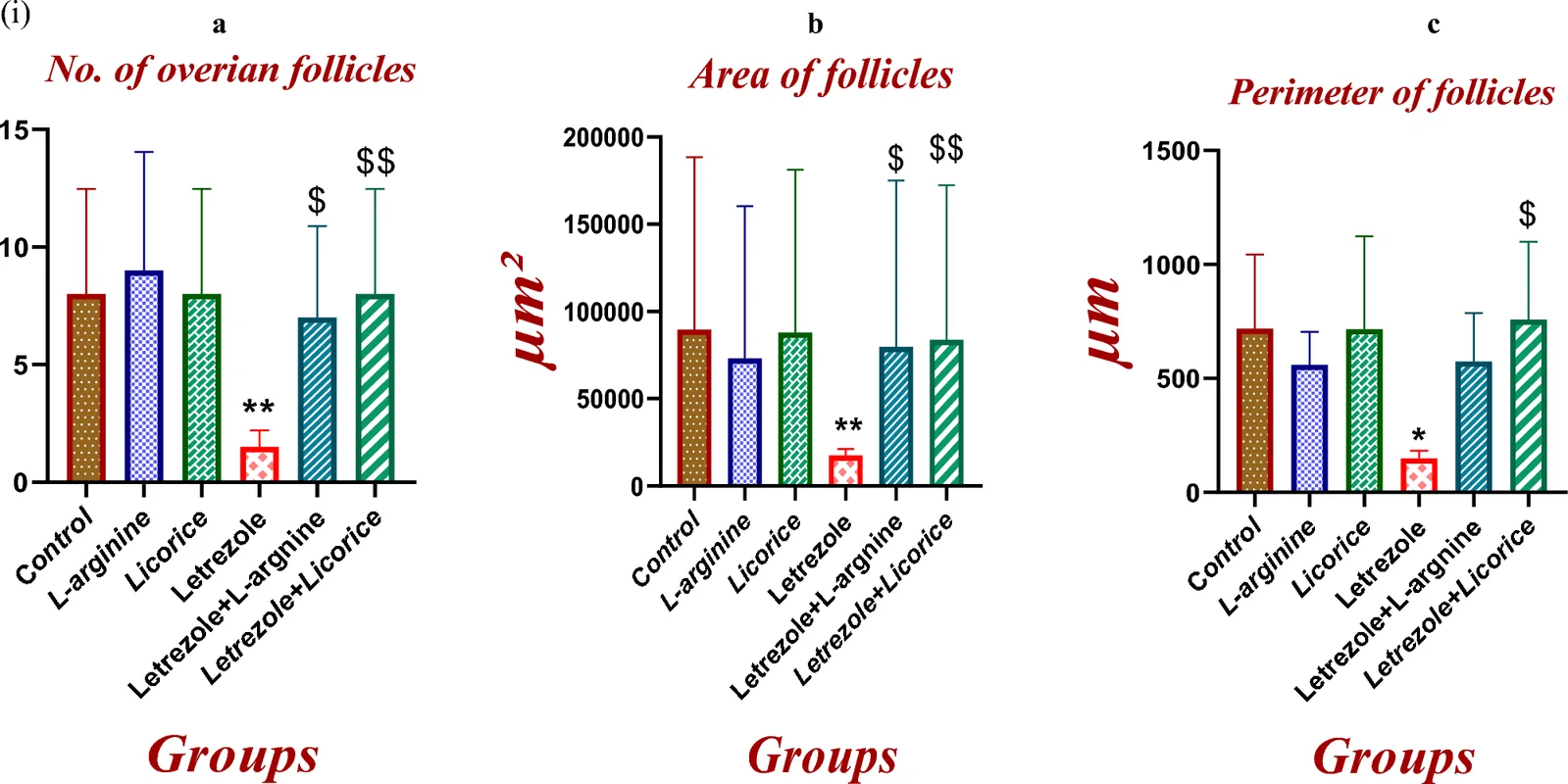
The ovarian sections of the different animal groups showing (**a**), No. of ovarian follicles, (µm^2^; **b**), Area of ovarian follicles & (µm; **c**), Perimeter of follicles in different rat groups. Data are shown as mean ± SEM (*n* = 6). ^*, $^ Significant at *P* < *0.05* and ^**, $$^ Significant at *P* < *0.01.* ^*, **^ Indicate comparisons with respect to the control group. ^**$, $$**^ Indicate comparisons with respect to the PCOS group. *Cont*: control, *Letrozole:* PCOS, *Letrozole* + *L-arginine:* PCOS + L-arginine, *Letrozole* + *Licorice:* PCOS + Licorice.

**Fig.10:**
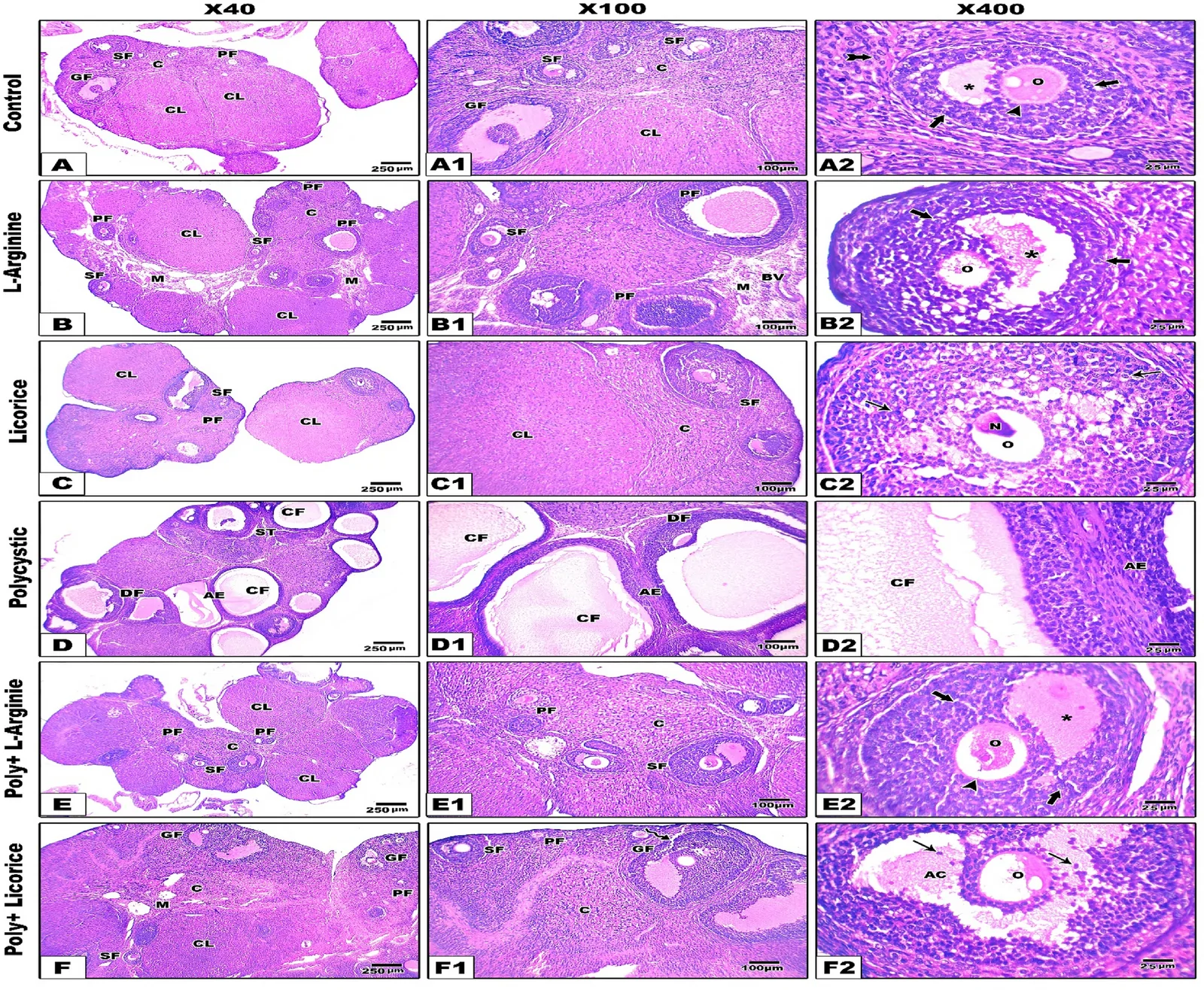
Histological changes in the ovarian sections of the different animal groups. (Fig. 10); **A** An ovary section of control group showing primary follicles (PF), secondary follicles (SF), graffian follicle (GF), and corpus luteum (CL) , **B** L-arginine group showing primary follicles (PF), secondary follicles (SF), and corpus luteum (CL) , **C** Licorice group showing primary follicles (PF), secondary follicles (SF), and corpus luteum (CL), **D** Section of ovary from a Letrozole (PCOS) group showing cystic follicles (CF), **E** Section of ovary from Letrozole + L-arginine group showing primary follicles (PF), secondary follicles (SF), and corpus luteum (CL) & **F** Section of ovary from Letrozole + Licorice group showing primary follicles (PF), secondary follicles (SF), graffian follicles (GF), and corpus luteum (CL).

Histopathological assessment of ovarian structure was conducted using hematoxylin and eosin staining (Fig. 10). The control group, as well as the L-arginine or licorice extract-treated groups, exhibited normal ovarian structures, including well-developed oocytes and ovarian follicles with normal oocyte number, normal follicular area and perimeter (*P* ≤ 0.01, *P* ≤ 0.01, and *P* ≤ 0.05, respectively). In contrast, rats treated with letrozole displayed numerous cysts of varying sizes in their ovaries. However, rats treated with letrozole in combination with L-arginine or licorice extract demonstrated ovarian structures similar to the control group, including normal follicular development and the absence of the multiple cysts observed in the untreated PCOS group (Figs. 9 and 10).

**Fig. 11:**
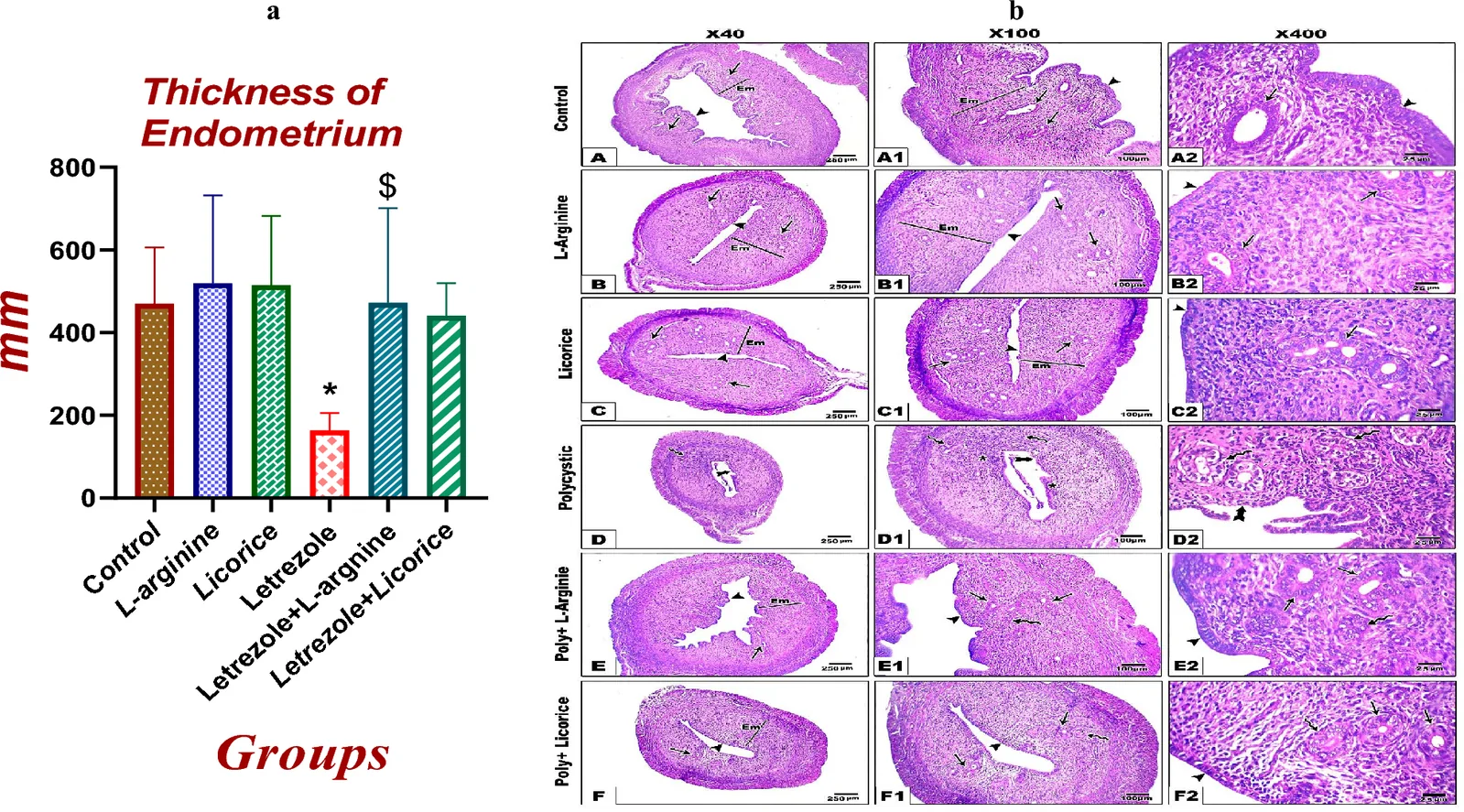
Photomicrographs showing the effect of L-arginine & Licorice on various animal groups’ thickness of endometrium (mm; a), in uterine sections. Data are shown as mean ± SEM (*n* = 6). ^*, $^ Significant at *P* < *0.05*. ^*^ Indicate comparisons with respect to the control group. ^**$**^ Indicate comparisons with respect to the PCOS group. *Cont*: control, *Letrozole:* PCOS, *Letrozole* + *L-arginine:* PCOS + L-arginine, *Letrozole* + *Licorice:* PCOS + Licorice. Histological uterine sections in the various animal groups. (Fig. 11b); **A** control, **B** L-arginine, **C** Licorice, **D** Letrozole (PCOS), **E** Letrozole + L-arginine & **F** Letrozole + Licorice groups showing Endometrium (Em), activated columnar epithelium (arrowhead), normal endometrial glands (arrow ↓), atrophy of the endometrium with marked loss of endometrial glands (wavy arrow ), hyperplasia on surface epithelium (asterisk *), and detached epithelium of endometrium (thick arrow ).

Histopathological analysis of the uterine structure was performed using hematoxylin and eosin staining (Fig. 11b). Normal uterine structure was observed in the control group and the groups treated with L-arginine or licorice extract. The uterus was surrounded by a thick layer of smooth muscle known as the myometrium and bordered by a thick mucosa called the endometrium. The endometrium was a dense vascular stroma supporting a simple columnar epithelium that formed numerous tubular glands. In contrast, polycystic rats exhibited atrophied uterine endometrium with a notable reduction in endometrial glands and hyperplasia of the surface epithelium. Polycystic rats treated with L-arginine or licorice extract demonstrated restored endometrial thickness and an increased number of endometrial glands (Fig. 11a-b).

**Fig.12:**
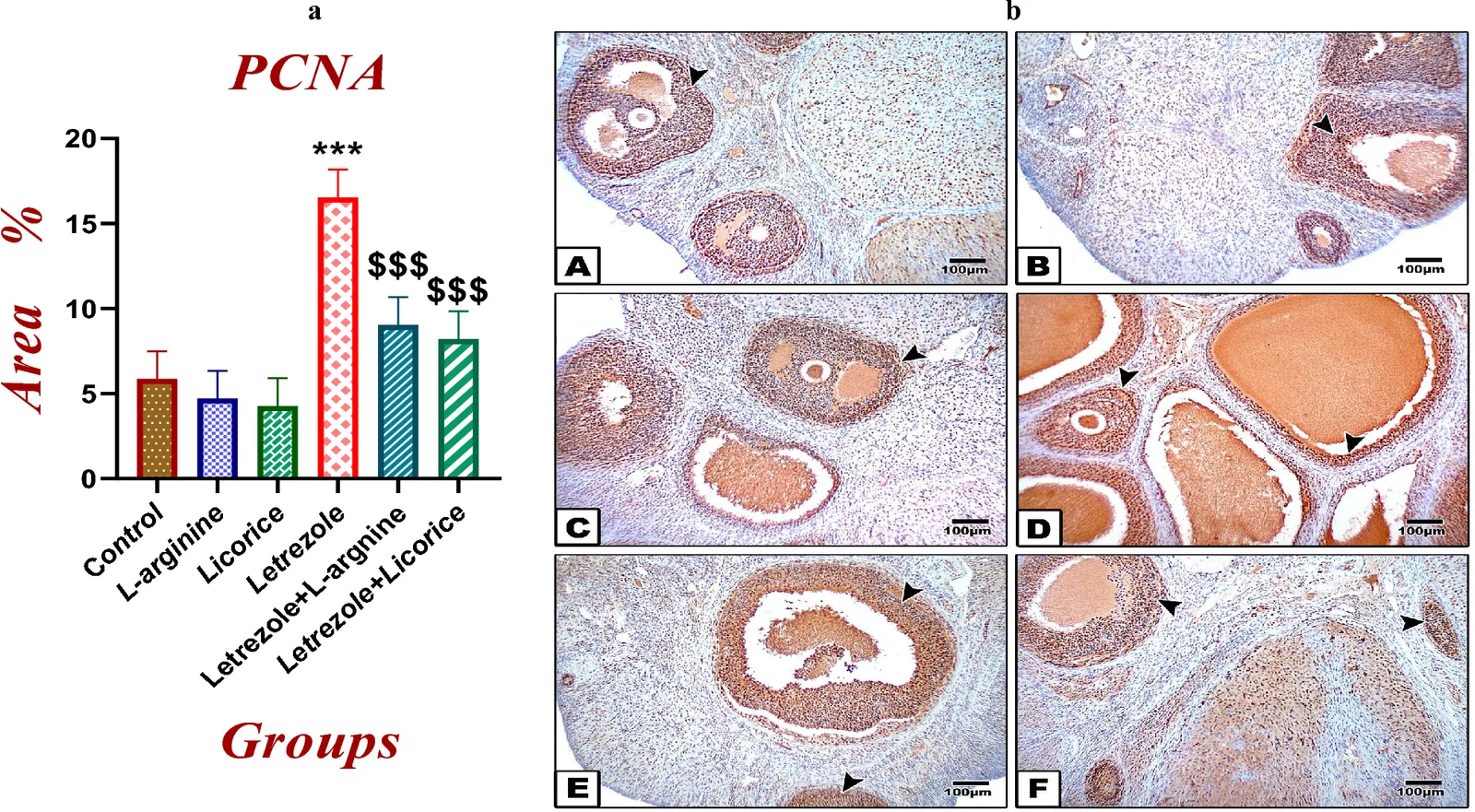
Quantitative analysis showing the percentage area (%) of positive PCNA immunostaining (%; Fig. 12a) in ovarian sections in different rat groups. Data are shown as mean ± SEM (*n* = 6). ^***, $$$^ Significant at *P* < *0.001.* ^***^ Indicate comparisons with respect to the control group. ^**$$$**^ Indicate comparisons with respect to the PCOS group. *Cont*: control, *Letrozole:* PCOS, *Letrozole* + *L-arginine:* PCOS + L-arginine, *Letrozole* + *Licorice:* PCOS + Licorice. Immunity parameter levels. (Fig. 12b), PCNA levels in different rat groups: (A) control, (B) L-arginine, (C) Licorice, (D) Letrozole (PCOS), (E) Letrozole + L-arginine, (F) Letrozole + Licorice.

**Fig.13:**
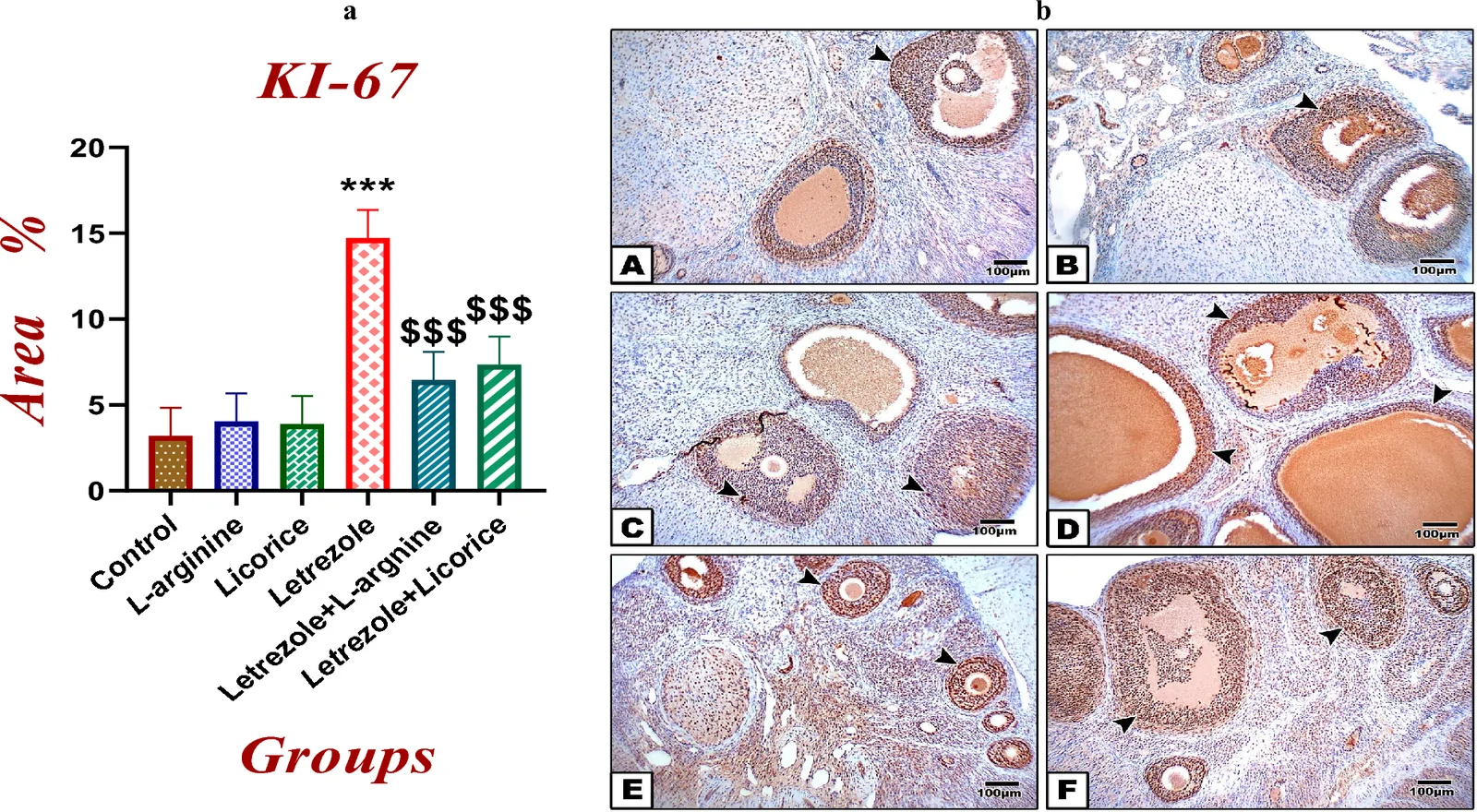
Quantitative analysis showing the percentage area (%) of positive Ki-67 immunostaining (%; Fig. 13a) in ovarian sections in different rat groups. Data are shown as mean ± SEM (n = 6). ^***, $$$^ Significant at P < 0.001. Immunity parameters levels (Fig. 13b), KI-67 levels in different rat groups; (**A**) control, (**B**) L-arginine, (**C**) Licorice, (**D**) Letrozole (PCOS), (**E**) Letrozole + L-arginine, (**F**) Letrozole + Licorice.

The proliferation index of ovarian granulosa cells was assessed using PCNA and Ki-67 markers through immunohistochemistry. PCNA and Ki-67 expression levels were significantly elevated in the PCOS group (*P* ≤ 0.001) compared to the control group (Figs. 12a–b, 13a-b). However, in polycystic rats treated with L-arginine or licorice extract, the expression of PCNA and Ki-67 was significantly reduced compared to the untreated PCOS group (Figs. 12a-b, 13a-b).

**Fig.14:**
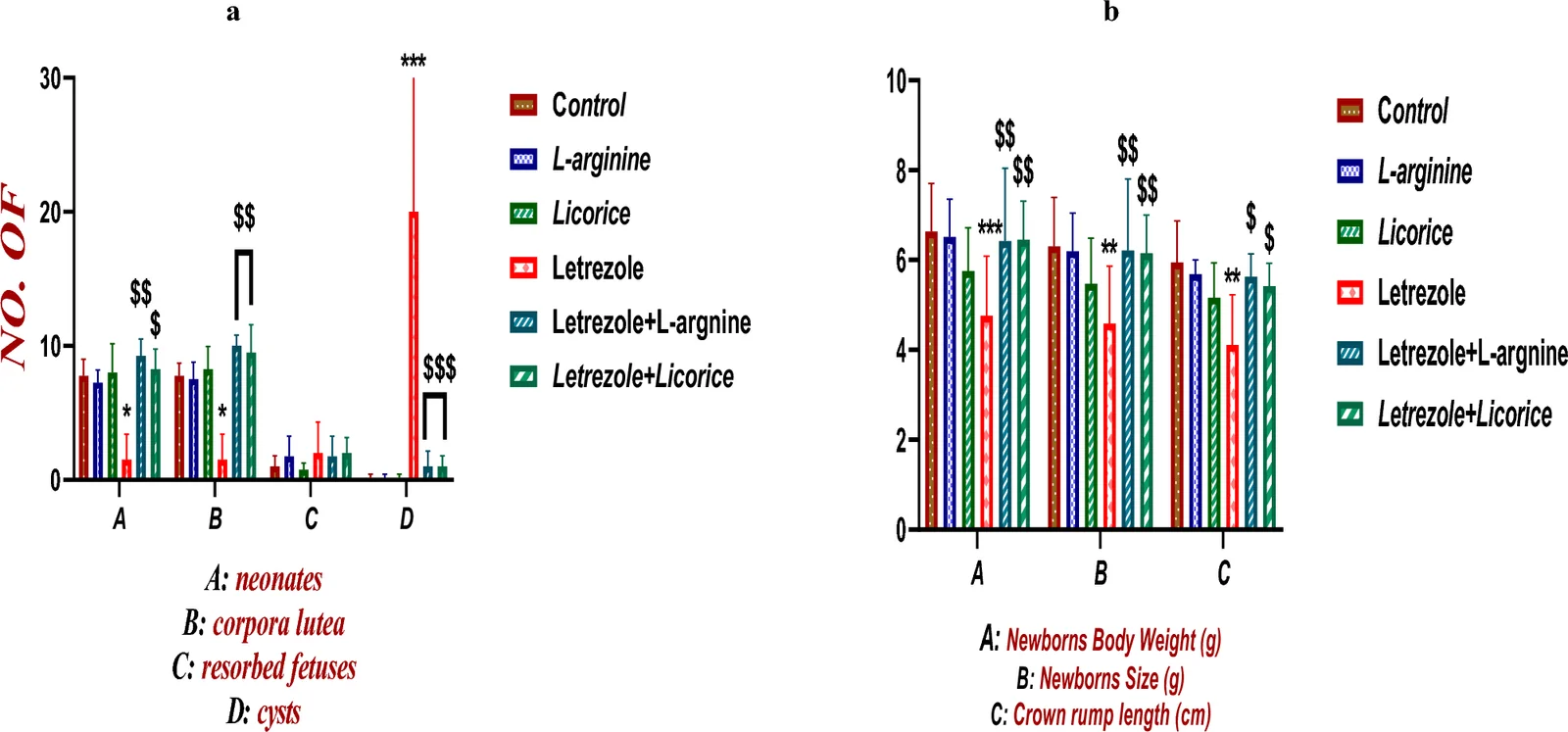
Effect of Letrozole, L-arginine & Licorice on Fertility index. (**a**), A: neonates, B: corpora lutea, C: resorbed fetuses, D: cysts & (**b**), A: Newborns Body Weight (g), B: Newborns Size (g), C: Crown rump length (cm) in different rat groups. Data are shown as mean ± SEM (*n* = 6). ^*, $^ Significant at *P* < *0.05*, ^**, $$^ Significant at *P* < *0.01* and ^***, $$$^ Significant at *P* < *0.001.* ^*, **, ***^ Indicate comparisons with respect to the control group. ^**$, $**^ Indicate comparisons with respect to the PCOS group. *Cont*: control, *Letrozole:* PCOS, *Letrozole* + *L-arginine:* PCOS + L-arginine, *Letrozole* + *Licorice:* PCOS + Licorice.

Tables 2 and 3, along with Figs. 14a–b, summarize the fertility indices of the control and experimental groups. The findings show that all four rats in the control, L-arginine, or licorice extract groups gave birth within 21–25 days, with mean litter sizes of 7.750 ± 0.629, 7.250 ± 0.479, and 8.000 ± 1.080, respectively. In contrast, in the PCOS group, only 2 out of 6 rats gave birth, and this occurred after a significantly delayed period of 50–60 days. The mean litter size (1.500 ± 0.957, *P* < 0.05) and mean pup body weight (4.765 ± 0.540, *P* < 0.001) in the PCOS group were significantly lower than those in the control group. No visible abnormalities were noted in the offspring. The remaining four rats in the PCOS group did not reproduce during the two-month observation period.

**Table.2:** Fertility indexes results for the control and other experimental groups.

Animal groups (n/group = 4)		Control	L-arginine	Licorice	Letrozole (PCOS)	Letrozole + L-arginine	Letrozole + Licorice
No. of mated rats		4	4	4	6	4	4
No. of breeding rats (impregnated)		4	4	4	2	4	4
% Fertility success [no. of females impregnated / no. of females mated] (%)		4/4 (100%)	4/4 (100%)	4/4 (100%)	2/6 (33.3%)	4/4 (100%)	4/4 (100%)
Mating length (A) (days)		25	24	23	60	31	29
Maternity period [A-21] (days)		4	3	2	39	10	8
Total no. of neonates (range)		27 (8–10)	25 (8–10)	32 (8–11)	6 (0–6)	35 (8–12)	32 (8–11)
Mean litter size [total no. of neonates/ group]	Mean ± SE	7.750 ± 0.629	7.25 ± 0.479	8.000 ± 1.080	1.500 ± 0.957*	9.250 ± 0.629^$$^	8.250 ± 0.750^$^
No. of corpora lutea	Mean ± SE	7.750 ± 0.479	7.5 ± 0.645	8.250 ± 0.854	1.500 ± 0.957*	10.00 ± 0.408^$$^	9.500 ± 1.041^$$^
No. of resorbed fetuses	Mean ± SE	1.00 ± 0.408	1.75 ± 0.750	0.750 ± 0.250	2.000 ± 1.155	1.750 ± 0.750	2.000 ± 0.577
No. of cysts	Mean ± SE	0.00 ± 0.00	0.00 ± 0.00	0.00 ± 0.00	20.00 ± 6.258***	1.000 ± 0.577^$$$^	1.000 ± 0.408^$$$^

♦Values are presented as Mean ± SEM, n = 4, *P < 0.05 and ***P < 0.001 compared to control group, ^$^*P* < 0.05, ^$$^*P* < 0.01 and ^$$$^*P* < 0.001 compared to PCOS group.

**Table.3:** Newborns’ body weight, Size (g) and Crown rump length (CRL) (cm) for the control and other experimental groups.

		Cont	L-arginine	Licorice	Letrozole (PCOS)	Letrozole + L-arginine	Letrozole + Licorice
Newborn’s body weight	Mean ± SE	6.641 ± 0.213	6.512 ± 0.175	5.752 ± 0.178	4.765 ± 0.540***	6.417 ± 0.284^$$^	6.455 ± 0.157^$$^
% of change	*		−1.94	−13.4	−28.2	−3.37	−2.80
	**					+ 34.7	+ 35.5
Newborns Size	Mean ± SE	6.300 ± 0.220	6.196 ± 0.177	5.467 ± 0.187	4.583 ± 0.523**	6.212 ± 0.278^$$^	6.150 ± 0.156^$$^
% of change	*		−1.65	−13.2	−27.3	−1.40	−2.38
	**					+ 35.5	+ 34.2
Crown rump length (CRL)	Mean ± SE	5.9496 ± 0.185	5.687 ± 0.066	5.166 ± 0.141	4.117 ± 0.456**	5.630 ± 0.088^$^	5.417 ± 0.094^$^
% of change	*		− 4.41	−13.17	−30.8	−5.37	−8.95
	**					+ 36.8	+ 31.6

♦Values are presented as Mean ± SEM, n = 4.

**P < 0.01 and ***P < 0.001 compared to control group, ^$^*P* < 0.05 and ^$$^*P* < 0.01 compared to PCOS group, (*) and (**) are the percentage of change compared to the control and PCOS groups, respectively.

All four rats in the PCOS groups treated with L-arginine or licorice extract gave birth within 29–31 days. These treatments resulted in significantly increased mean litter sizes of 9.250 ± 0.629 (*P* < 0.01) and 8.250 ± 0.750 (*P* < 0.05), respectively, compared to the untreated PCOS group. Similarly, the mean pup body weights were significantly higher, measuring 6.417 ± 0.284 (*P* < 0.01) in the L-arginine group and 6.455 ± 0.157 (*P* < 0.01) in the licorice extract group. Additionally, the offspring exhibited healthier body conditions with no visible malformations.

“Mating experiments were performed with four females from each group, except for the PCOS group, where six females were used due to the expected reduced fertility.”

## Discussion

Polycystic ovarian syndrome (PCOS) is a common endocrine disorder affecting women of reproductive age, characterized by polycystic ovaries, hyperandrogenism, and chronic anovulation. Beyond fertility issues, PCOS is linked to comorbidities such as obesity, metabolic syndrome, type 2 diabetes, and endometrial cancer^10^.

Increasing interest in natural products for PCOS management has emerged due to their bioactive compounds and minimal side effects. One such product, L-arginine, a semi-essential amino acid found in meats and nuts, is a precursor for nitric oxide (NO). NO is recognized for its diverse roles as an intra- and intercellular signaling molecule, influencing various physiological systems, including ovarian function. Although the precise mechanisms remain unclear, NO is believed to contribute to critical processes such as follicular maturation and ovulation^11^.

Licorice, a widely used medicinal herb, contains bioactive compounds like glycyrrhizin and flavonoids, known for their antioxidative, antifatigue, antibacterial, antiviral, antiproliferative, and estrogenic properties. These properties have sparked interest in licorice as a potential therapeutic for conditions like polycystic ovary syndrome (PCOS)^5^.

In PCOS, lipid metabolism disturbances are a prominent feature, with altered lipid profiles showing elevated cholesterol, triglycerides, and low-density lipoprotein (LDL) levels, along with reduced high-density lipoprotein (HDL) levels. These lipid changes, particularly the reduction in HDL-C and elevation of plasma triglycerides, are consistent with findings by Ibrahim et al*.*^12^, who reported similar changes in obese women with PCOS. This dyslipidemia is associated with an increased cardiovascular risk in individuals with PCOS, highlighting the importance of managing lipid levels in this condition.

Supplementation with L-arginine or licorice extract significantly improved lipid profiles in PCOS, increasing HDL and reducing cholesterol, triglycerides, and LDL. These results are consistent with studies by Hadi et al*.*^13^, which linked L-arginine’s lipid-enhancing effects to its ability to boost nitric oxide (NO) production, improving lipid dynamics and regulating vascular health. Similarly, Alwash et al*.*^14^, found that licorice supplementation improved lipid parameters, likely due to the antioxidant and hypolipidemic properties of *Glycyrrhiza glabra*^15^. The observed lipid-lowering effects of licorice may be defined by the presence of bioactive compounds such as phytosterols and saponins in its roots. Phytosterols are well-documented for their ability to reduce serum cholesterol by inhibiting intestinal cholesterol absorption. Additionally, saponins interact with bile acids and cholesterol to form insoluble complexes, thereby preventing their reabsorption in the gastrointestinal tract. This mechanism promotes the elimination of cholesterol through fecal excretion, contributing to the overall improvement in lipid profile^14^.

The use of L-arginine and licorice extract in PCOS rats resulted in a decrease in serum lipid parameters, such as LDL, and an increase in HDL. However, care should be taken when interpreting changes in HDL and LDL in rodent models, since lipid metabolism is quite different in rodents and humans. Rodents have HDL-dominant lipoprotein profiles and low cholesteryl ester transfer protein (CETP) activity, leading to lipoprotein distributions not identical to human physiology. Thus, even though the observed alterations in lipids are good markers of metabolic enhancement in the experimental model, direct extrapolation to human cardiovascular risk should be cautiously performed^16^.

In PCOS, oxidative stress markers like malondialdehyde (MDA) and nitric oxide (NO) are elevated, while antioxidants like catalase and superoxide dismutase (SOD) are reduced, confirming findings by Tefagh et al*.*^17^, who highlighted that PCOS is characterized by a significant reduction in antioxidant levels and an increased susceptibility to oxidative stress. This imbalance between oxidative stress and antioxidant defenses is a critical factor in the pathophysiology of PCOS. The imbalance of oxidative stress (OS) within the follicular fluid environment has profound implications for reproductive outcomes. Elevated oxidative stress can impair oocyte development, hinder proper embryonic growth, and lead to suboptimal pregnancy outcomes^18^.

Administration of L-arginine or licorice extract in PCOS significantly reduced oxidative stress markers like MDA and NO, while increasing antioxidant markers such as catalase and SOD. These outcomes are consistent with findings from previous research^19,20^. According to Liang et al*.* ^19^, demonstrated that L-arginine supplementation enhances glutathione (GSH) synthesis and activates the Nrf2-Keap1 pathway, leading to the upregulation of antioxidant response element (ARE)-driven antioxidant expressions. This highlights the critical role of L-arginine in mitigating oxidative stress and triggering endogenous antioxidant defenses. Similarly, the results align with those of Ojha et al*.* ^20^, who reported that licorice provides cardioprotective effects by reducing oxidative stress, increasing endogenous antioxidant levels, restoring functional parameters, and preserving structural integrity. These findings underscore the strong antioxidant and hypolipidemic effects of licorice and its components, which have been extensively studied for their bioactive properties^14,15^.

In the current investigation, we found that the impact of licorice extract on catalase activity was context-dependent. The lack of excessive oxidative stress necessitating enzymatic upregulation may be the reason why licorice did not significantly change catalase levels in healthy control rats. On the other hand, antioxidant defenses, including catalase, are frequently compromised in the letrozole-induced PCOS model, where oxidative stress is known to be elevated and can be improved by treatment with antioxidant compounds. This context-dependent modulation probably reflects licorice bioactive constituents’ capacity to support endogenous antioxidant mechanisms and counteract reactive oxygen species in pathological conditions, improving redox balance in tissues affected by PCOS. The role of oxidative stress and dysregulated antioxidant enzyme activities, such as catalase, in PCOS-induced animals has been highlighted in earlier research. In experimental models, oxidative imbalance contributes to the pathogenesis of the disease and can be mitigated by antioxidant treatments^21^.

In addition to oxidative stress, PCOS also increased inflammatory markers, including tumor necrosis factor-alpha (TNF-α) and interleukin-6 (IL-6). These results are consistent with the findings of Dey et al*.*^22^, who identified obesity and adipocytes as key contributors to the production of pro-inflammatory mediators, resulting in chronic low-grade inflammation. Given the close association between PCOS, insulin resistance, and obesity, the elevated levels of inflammatory markers may further exacerbate the metabolic and reproductive disturbances seen in PCOS.

Supplementation with L-arginine or licorice reduced these inflammatory markers (TNF-α and IL-6), thereby improving the inflammatory profile. These findings are consistent with previous studies, such as those by Qiu et al*.*^23^, who demonstrated that L-arginine exhibits both antioxidant and anti-inflammatory properties, effectively protecting cells from oxidative stress and inflammatory responses. The ability of L-arginine to modulate inflammation further highlights its potential as a therapeutic agent in managing PCOS-related inflammation. Additionally, the results align with the findings of Yu et al*.*^24^, who reported that licorice significantly reduced the production of pro-inflammatory cytokines, including TNF-α, IL-1β, and IL-6. Licorice (Glycyrrhiza glabra), a widely used medicinal herb with a long history in traditional medicine, is known for its diverse pharmacological properties, its anti-inflammatory, antibacterial, antioxidant, and antiviral activities^5^.

The current results supported antioxidant and anti-inflammatory effects of L-arginine and licorice extract, and these beneficial effects observed in the study may involve signaling pathways previously implicated in PCOS pathophysiology, but did not directly assess the significance of upstream signaling pathways, including Nrf2-mediated antioxidant responses, NF-κB-associated inflammatory regulation, and PI3K/Akt signaling. Moreover, mechanical investigations are needed to clarify the possible molecular pathways involved in the observed protective effects.

The study highlights significant hormonal alterations in PCOS, including elevated LH, testosterone, and AMH levels, alongside reduced FSH and estradiol (E2). These findings are consistent with Abbas et al*.*^25^, which attributes PCOS to aberrant ovarian androgen production leading to hyperandrogenism. The hormonal imbalance observed in PCOS underpins a range of clinical manifestations, including menstrual irregularities, polycystic ovarian morphology, and hyperandrogenism, evidenced through clinical or laboratory markers. Additionally, these alterations contribute to disrupted fertility, elevated insulin levels, and a characteristic imbalance between LH and FSH. Also, the study revealed that PCOS significantly elevated AMH levels, a finding consistent with Lie Fong et al*.*^26^, who suggested serum AMH levels as a diagnostic marker for PCOS. Elevated AMH levels are attributed to the increased number of pre-antral and antral follicles in polycystic ovaries, which further upregulate AMH synthesis^27^. Higher AMH levels are particularly useful for assessing ovarian volume and diagnosing PCOS, as they reflect the extent of follicular dysregulation characteristic of the condition.

Treatment with L-arginine or licorice extract significantly increased FSH and estradiol while decreasing LH, testosterone, and AMH in PCOS rats. These results are in line with Ragy et al*.*^28^, who reported that L-arginine mitigates PCOS symptoms due to its antioxidant and anti-inflammatory effects through nitric oxide (NO), which regulates follicular growth and ovulation, and luteal function in the ovaries^29^. L-arginine appears to restore hormonal balance, improving fertility, thus alleviating PCOS-related symptoms. Similarly, the findings align with those of Yang et al*.*^30^, who highlighted the effectiveness of licorice extract in managing PCOS symptoms by correcting hormonal imbalances, reducing ovarian cysts and volume, enhancing insulin sensitivity, normalizing reproductive cycles, and improving hormonal profiles.

In the current study, administering licorice extract or L-arginine to normal rats was linked to slight increases in AMH and testosterone levels. Under physiological conditions, where endocrine homeostasis is maintained, and temporary stimulation of steroidogenesis can occur without pathological consequences, this effect might be the result of mild modulation of basal steroidogenic pathways. On the other hand, both L-arginine and licorice extract had regulatory rather than stimulatory effects in the hyperandrogenic PCOS model, which resulted in lower levels of AMH and testosterone. The capacity of both agents to regulate oxidative stress, inflammation, and ovarian steroidogenesis in the pathological PCOS environment may be the cause of this different reaction. Antioxidant or redox-modulating treatments can restore hormonal balance by reducing excessive androgen synthesis, according to mounting evidence that oxidative stress is a key factor in dysregulated steroidogenesis and elevated androgen production in PCOS. Therefore, the complex interaction between oxidative stress and endocrine regulation in PCOS is consistent with the observed dual and context-dependent hormonal effects^3,21^.

The study showed that PCOS significantly increased insulin-like growth factor-1 (IGF-1) and insulin hormone levels. These findings are in agreement with previous studies^31,32^. El-Hassanin et al*.*^31^, reported elevated circulating IGF-1 levels in PCOS patients compared to healthy controls, suggesting that IGF-1 could serve as a potential biochemical marker for diagnosing PCOS. Similarly, Singh et al*.*^32^, highlighted that 65–70% of women with PCOS experience insulin resistance accompanied by compensatory hyperinsulinemia. Hyperinsulinemia appears to play a multifaceted role in PCOS pathophysiology. It acts as a co-gonadotropin, directly stimulating theca cells to produce excess androgens, while also exacerbating hyperandrogenemia by amplifying the effects of elevated LH levels observed in most individuals with PCOS^33^.

Treatment with L-arginine or licorice extract significantly reduced insulin hormone and IGF-1 levels in PCOS rats, supporting the findings of Rad et al*.*^34^, which demonstrated that L-arginine is a safe and effective compound for reducing serum insulin levels and fasting blood glucose in individuals with glucose metabolism disorders. L-arginine improves insulin sensitivity and glucose metabolism while reducing insulin resistance, making it a valuable therapeutic agent for managing metabolic dysfunctions associated with PCOS. Additionally, our findings align with those of Hooshmandi et al*.*^7^, who reported that licorice root supplementation can improve body composition and mitigate insulin resistance. These effects further highlight the potential of licorice extract as a complementary treatment for metabolic abnormalities in PCOS.

In the present study, serum insulin levels were analyzed, but additional measurements such as fasting blood glucose and HOMA-IR should be included in future studies to give a more complete evaluation of insulin resistance.

PCOS also significantly increased the levels of Caspase-3 and P53 in the control group. These findings are consistent with those reported ^**^Corresponding authors: aboulnaga@mans.edu.eg and amoura55555@gmail.com. highlighted the involvement of the Bax/Bcl-2/P53 and Caspase-3 (Cas-3) pathway in the pathophysiology of PCOS. This pathway plays a critical role in androgen modulation, steroidogenesis in theca and granulosa cells, and the development of dominant follicles, while also contributing to enhanced mitochondrial apoptosis of granulosa cells. Insulin resistance and hyperinsulinemia, which are hallmark features of PCOS, can dysregulate the expression of genes associated with apoptosis.

Administration of L-arginine or licorice extract to PCOS rats significantly decreased the levels of Caspase-3 and P53, suggesting a reduction of apoptotic activity. These findings are consistent with previous studies^36,37^. Chalisova et al*.*^36^ demonstrated that arginine effectively reduced the area of P53 expression in explants of both mature and immature spleen tissue, highlighting its potential to modulate P53 expression. While this mechanism has been linked to tumor growth promotion, it also points to arginine’s ability to modulate apoptotic pathways. Similarly, Zheng et al*.*^37^ found that L-arginine significantly reduced caspase-3 activity induced by LPS and decreased caspase mRNA expression at multiple time points, underscoring its anti-apoptotic properties. The findings for licorice extract are also consistent with previous research ^38,39^. Ju et al*.*^38^ reported that glycyrrhizic acid and licorice extract reduced reactive oxygen species (ROS) formation and P53 phosphorylation in cells exposed to cisplatin, highlighting their antioxidant and anti-apoptotic properties. Mobasher et al*.*^39^, demonstrated that glycyrrhetinic acid, a key component of licorice, suppresses caspase-3 and TNF-α, contributing to its hepatoprotective effects.

Our study’s apparent contradiction in apoptotic marker expression is probably due to the treatments’ context-specific biological effects. A slight rise in caspase and p53 levels in healthy ovarian tissue may indicate typical physiological cell turnover and homeostatic control. On the other hand, excessive pathological apoptosis caused by oxidative stress and hormonal imbalance characterizes the ovaries in the PCOS model. A decrease in caspase and p53 expression was linked to treatment with L-arginine and licorice extract in this situation, indicating a protective, anti-apoptotic effect that might be mediated by antioxidant and cytoprotective mechanisms. This explanation confirms the idea that apoptotic pathways react differently in healthy and pathological circumstances^40,41^.

In this study, PCOS significantly increased Amhr2 gene expression. These outcomes are consistent with the findings of Bhimwal et al.^42^, who observed that, unlike normal ovulatory females who exhibit no AMH gene expression during follicle maturation, women with PCOS display increased follicle counts, elevated AMH levels per follicle, and upregulated expression of AMH and AMHR2. These alterations are indicative of disrupted follicular maturation and the persistence of immature follicles, a hallmark of PCOS.

Interestingly, the administration of L-arginine or licorice extract significantly reduced Amhr2 gene expression in PCOS rats, suggesting a potential regulatory effect on this pathway. Uyanga et al.^43^ reported that arginine supplementation influences ovarian morphology and modulates the expression of reproductive hormone-related genes in the hypothalamic-pituitary–gonadal axis. These effects are likely mediated through mechanisms involving nitric oxide (NO) and insulin-like growth factor-1 (IGF-1), both of which are known to influence ovarian function. This may explain the observed effects of L-arginine on Amhr2 expression. For licorice extract, Shamsi et al.^44^ found that oral licorice extract consumption can significantly lower testosterone levels while improving oocyte maturation, fertilization rates, and embryo development in mice. Furthermore, licorice extract has been shown to enhance ovarian morphology in animals with PCOS. These findings align with the current study and suggest that licorice extract’s impact on hormonal regulation and ovarian health may also contribute to the observed reduction in Amhr2 gene expression.

Although Amhr2 expression is highly correlated with follicular development and ovarian function, the mechanistic role of Amhr2 in mediating the therapeutic effects observed in this study remains unknown. Therefore, the current findings should be interpreted as associative rather than causal, and future studies are required to investigate the downstream signaling pathways involved.

Histopathological analysis revealed that PCOS induced numerous cystic formations characterized by cystic atretic follicles. These observations are consistent with previous research. Shamsi et al.^44^ reported a significant increase in atretic follicles and a notable decrease in the number of healthy follicles in the corpus luteum (CL) of PCOS-affected ovaries. The elevated testosterone levels observed in the PCOS group are a key factor contributing to these histopathological changes. Hyperandrogenism disrupts the normal follicular development process, leading to the formation of cystic follicles, degeneration of granulosa cells, and an increased prevalence of atretic follicles.

L-arginine and licorice extract administration reduced cystic follicles, promoting healthy follicular stages. These findings are in line with Ragy et al.^28^, who suggested that L-arginine, through its conversion to nitric oxide (NO), may help reduce serum testosterone levels by inhibiting steroidogenesis via the binding of NO to cytochrome P-450, an enzyme necessary for testosterone synthesis. NO, as a vasodilator, also promotes increased ovarian blood flow, which is critical for normal ovulation. These results are consistent with the findings of Shamsi et al.^44^, who demonstrated that licorice extract significantly increased the number of healthy follicles and corpus luteum (CL) structures while reducing the number of atretic follicles in PCOS rats. Licorice extract has been shown in multiple studies to lower intra-ovarian androgen levels, thereby reducing androgen synthesis from estrogen. This reduction in androgen levels likely helps restore the hormonal balance and mitigate the hyperandrogenism associated with PCOS, which, in turn, may contribute to improved follicular development and a reduction in cystic formations.

PCOS also causes uterine abnormalities, including surface epithelium hyperplasia, localized necrosis, and uterine endometrial atrophy, along with a significant loss of endometrial glands. These findings are consistent with Haslan et al.^45^, who reported similar uterine changes, including decreased uterine weight and length, reduced endometrial thickness, and a lower number of endometrial glands. These alterations were associated with elevated levels of LH and testosterone and decreased levels of FSH, all of which are characteristic of PCOS. These observations provide further evidence supporting the notion that changes in gonadal morphology and reproductive hormone imbalances play a critical role in the uterine pathology observed in PCOS. Endocrinological evidence suggests that women with PCOS have an endometrium that differs significantly from that of healthy women, with a higher incidence of hyperplasia and an increased risk of developing endometrial cancer.

The treatment with L-arginine or licorice extract normalized endometrial thickness and increased the number of endometrial glands in PCOS rats. These findings are consistent with Takasaki et al.^46^, who observed that L-arginine promotes endometrial development by enhancing blood flow in the uterine radial arteries. The results suggest that improved uterine radial artery blood flow plays a key role in promoting endometrial growth. Additionally, these findings align with Tanideh et al.^47^, who found that licorice root extract significantly improved both biochemical parameters and histological markers of uterine tissue. This effect is likely attributed to the high concentrations of phytoestrogens in licorice, which have chemical structures similar to estradiol and can exert estrogenic effects on uterine tissue. The ability of licorice extract to enhance endometrial health in PCOS may therefore be linked to its modulation of estrogenic activity, supporting uterine tissue growth and function.

PCOS also significantly elevated proliferation markers PCNA and Ki-67. These findings are consistent with Abdallah et al.^48^, who reported heightened Ki-67 expression in granulosa cells and theca interna cells in PCOS. In the PCOS group, there was an increased rate of cell growth in the theca interna, which is often indicative of disrupted follicular development. The increased expression of these proliferation markers aligns with Lombardi et al.^49^, who suggested that interstitial cells, which are derived from the highly proliferative theca interna, play a crucial role in maintaining the hyperandrogenic state in PCOS by producing androgens. This suggests that aberrant cell proliferation contributes to both follicular dysregulation and hormonal imbalance in PCOS.

After treatment with L-arginine or licorice extract, the levels of PCNA and Ki-67 significantly decreased, indicating a reduction in cell proliferation. These results are consistent with Li et al.^50^, who found that L-arginine supplementation inhibited early cell proliferation in the glomerulus. In their study, L-arginine reduced the number of PCNA-positive nuclei, and further Western blot analysis confirmed a decrease in PCNA expression in glomerular proteins. Similarly, the findings of Deng et al*.*^51^, support the current results, as they observed that treatment with glycyrrhizin – either alone or in combination with cisplatin – reduced PCNA expression, restored body weight, and protected against liver and kidney damage in tumor-bearing mice. These studies indicate that either L-arginine or licorice extract may help modulate excessive cell proliferation, which is often seen in PCOS-related ovarian dysfunction.

Besides the favorable results observed in hormonal, oxidative stress, and reproductive parameters, body weight alterations during the study were previously described by our group^52^. In brief, PCOS rats showed a significant body weight increase, and treatment with L-arginine or licorice extract significantly decreased body weight in comparison to untreated PCOS rats. Moreover, the whole reproductive tract, ovaries, and uterus weights were evaluated, and no clear abnormalities or obvious signs of toxicity were detected throughout the experimental duration. Nevertheless, detailed safety evaluation, including biochemical and electrolyte analysis, was beyond the scope of the current study and needs to be further investigated, particularly considering the recognized mineralocorticoid-like actions of licorice.

### Clinical relevance

The current research findings provide preclinical evidence for L-arginine and licorice extract as complementary approaches for PCOS. Particularly, a complementary clinical study on the evaluation of these interventions in women with PCOS has already been completed and is under preparation for publication. Additional clinical trials are needed to confirm efficacy and safety.

## Conclusion

The outcomes of the study propose that L-arginine or licorice extract may serve as effective therapeutic options for managing PCOS. Our results demonstrate that oral intake of L-arginine or licorice extract can significantly reduce testosterone levels, oxidative stress, and inflammatory markers, while simultaneously increasing estrogen levels and enhancing antioxidant activity. Moreover, these treatments showed notable improvements in oocyte maturation, fertilization, and embryo development rates in PCOS-induced rats. Additionally, L-arginine or licorice extract was observed to improve ovarian morphology and reduce the occurrence of ovarian cysts. These findings highlight the potential of these natural compounds for alleviating PCOS-related symptoms and restoring ovarian function.

### Limitations

Notwithstanding the thorough evaluation of metabolic, hormonal, molecular, and histopathological parameters, it is important to recognize the limitations of the current investigation. First, it is important to exercise caution when extrapolating the results to human disease because they are based on an experimental rat model of PCOS. The study’s main focus was on biochemical, molecular, and fertility-related outcomes rather than cyclicity itself, but the lack of estrous cycle monitoring is a limitation. While daily vaginal smear data were monitored during the pilot phase to confirm the 21-day letrozole induction protocol, systematic daily cytology records for all experimental animals were unfortunately not quantified in the final ledger. However, the successful induction of PCOS-like anovulation is strongly substantiated in our study by the hallmark histopathological findings (loss of primary/graafian follicles, presence of subcapsular cysts, and a depleted granulosa layer) alongside the highly significant drop in baseline fertility success (33.3%).

Additionally, long-term safety and toxicity evaluations were not conducted, especially for the licorice extract, and the intervention period was relatively short. Importantly, the present experimental study is part of a larger research project, and a corresponding clinical study has already been completed that evaluated the effects of L-arginine and licorice extract in women with PCOS, and the corresponding manuscript is being prepared for publication. The result needs to be confirmed and extended by further studies.

Another limitation of the current research is that a natural licorice extract was studied instead of isolated bioactives. The phytochemical profile of the botanical extracts might differ based on the cultivation and extraction conditions, and thus, further studies should take into account quantitative standardization of the major active compounds, such as glycyrrhizin and glabridin, to enhance reproducibility and facilitate clinical translation. However, herbal extracts consist of multiple phytochemicals that could exert synergistic or interactive biological activities, and accumulating evidence suggests that the overall therapeutic effects of licorice may be attributed to the combined actions of its constituents rather than a single compound. Thus, additional experiments to evaluate standardized extracts and individual bioactive components are required.

### Future perspectives

Future studies evaluating the combined administration of L-arginine and licorice extract may help determine whether additive or synergistic effects exist and further enhance their translational potential.

Further translational studies ought to consider the human equivalent dose conversion and dose optimisation.

Adding a standard pharmacologic control, such as metformin, might be very useful for evaluating efficacy and should be investigated in future studies.

## Materials and methods

## Ethical Approval:

The study protocol was approved by the Mansoura University Animal Care and Use Committee (MU-ACUC), Egypt, under approval number: MU-ACUC (SC.MS.23.05.24).

All experimental procedures involving animals were conducted in accordance with relevant institutional and national guidelines and regulations.

All methods are reported in accordance with ARRIVE guidelines (https://arriveguidelines.org).

Chemicals:

Letrozole and L-arginine were purchased from Sigma-Aldrich, Saint Louis, MO, USA.

Licorice was sourced locally from markets in Iraq.

Preparation of Licorice Extract:

1. Plant Material:

The dried licorice powder was purchased from the local market in Iraq. The expert Botanical Laboratory, Department of Botany, Faculty of Science, Mansoura University, Egypt, identified and authenticated the samples.

2. Extraction Process:

20 g of the dried Licorice powder was weighed and placed into a 250 mL conical flask.

100 mL of distilled water was added to the flask. The flask was placed in a horizontal water bath shaker at 70 °C for 30 min with shaking at 200 rpm. After extraction, the mixture was allowed to cool to room temperature.

3. Filtration:

The cooled extract was filtered using Whatman filter paper No. 1 (Whatman Int. Ltd., Kent, UK) through a Buchner funnel.

4. Concentration and Storage:

The concentration of the obtained extract was determined, and the solution was stored in sterile bottles at 0–5 °C under refrigerated conditions until further use^53^. The concentration of Licorice extract is 59.35 mg/ml.

Preparation of Licorice Ethanol Extracts for GC/MS Profiling only:

*Glycyrrhiza glabra* extract was obtained from the (Al-Ahliya Flavours & Fragrances Co. Ltd., Iraq)^54^. The Glycyrrhiza has been stored in a well-closed container protected from light and moisture^55^. 10 g of powder were mixed with 100 mL of 80% ethanol in a 250 mL conical flask and shaken for 2 h at room temperature. The flask was soaked and kept in the dark at 25 °C for 24 h, with occasional stirring. The mixture was then filtered, and the filtrate was stored in a cool, dark place for immediate use^56^.

### Experimental animals

In this investigation, 48 adult, healthy female Sprague Dawley (SD) rats, 6–7 weeks old and weighing an average of 150g, were utilized. The Egyptian Vaccine Company provided the animals used in this experiment (VACSERA, Giza, Egypt). For two weeks, the rats were left to acclimate to standard laboratory settings. They were housed in the animal house of the Zoology department, Faculty of Science, in stainless steel cages. The female rats were maintained under a 12-h light/dark cycle, with a stable temperature of 22–25 °C and humidity levels ranging between 50 and 60%. The study protocol was approved by the Mansoura University Animal Care and Use Committee, Egypt, with code NO. : MU-ACUC (SC.MS.23.05.24).

Each experimental group initially comprised twelve animals (n = 12). Following the induction and treatment periods, eight animals (n = 8) from each group were sacrificed for baseline biochemical, hormonal, molecular, histological, and morphometric analyses. The remaining four animals (n = 4) per group were reserved exclusively for mating experiments to evaluate and confirm fertility outcomes across the different study groups. However, in the untreated PCOS model group, six females were utilized for the mating assessment to account for the expected reduction in fertility rates.

### Experimental design and animal grouping

Following the acclimation period, the experimental animals were randomly and evenly divided into six groups (n = 12 rats per group) as follows:

1. *Control Group* Rats in this group received no treatment.
2. *Licorice Group* Rats were administered 150 mg/kg of licorice extract orally via a gastric tube daily for 21 days.
3. *L-Arginine Group* Rats received 22.9 mg/kg of L-arginine orally via a gastric tube daily for 21 days.
4. *PCOS Group* Polycystic Ovary Syndrome (PCOS) was induced in this group by administering 1 mg/kg of letrozole orally once daily for 21 days^57^.
5. *PCOS + Licorice Group* After the successful establishment of the PCOS model, rats were treated with 150 mg/kg of licorice extract orally via a gastric tube daily for 21 days^44^.
6. *PCOS + L-Arginine Group* After the successful establishment of the PCOS model, rats were treated with 22.9 mg/kg of L-arginine orally via a gastric tube daily for 21 days^58^

The doses of licorice extract (150 mg/kg/day) and L-arginine (22.9 mg/kg/day) were based on previous studies that showed efficacy and safety in experimental models. Translational context was provided by estimating the corresponding human equivalent doses (HEDs) using the FDA-recommended body surface area normalisation method. The HEDs calculated were approximately 24.3 mg/kg for licorice extract and 3.7 mg/kg for L-arginine, which equate to approximately 1.45 g/day and 222 mg/day, respectively, for a 60-kg adult.

### Sample collection and tissue preparation

#### Blood samples

At the end of the experiment, all rats were weighed, fasted overnight, and anesthetized with 75 mg/kg ketamine and 6 mg/kg xylazine via intraperitoneal injection. They were then euthanized by cervical dislocation. Animal welfare was prioritized through careful handling, enriched housing, monitoring for discomfort, and immediate interventions were implemented to alleviate distress when needed. All procedures were approved by the Ethics Committee for Laboratory Animals, Faculty of Science, Mansoura University.

After euthanasia, a midline inverted "T" incision exposed the heart, and 2–5 mL of blood was collected via cardiac puncture into plain tubes for clotting. The serum was separated by centrifugation at 5000 rpm for 5 min and stored at −20 °C for hormonal and biochemical analyses.

#### Tissue samples

Immediately after blood collection, the rats were dissected, and the ovaries and uterus were carefully removed. The organs were weighed, and the organ-to-body weight ratio was calculated. The uterus and left ovary were preserved in buffered formalin for histological and immunohistochemical investigations. The right ovary from each rat was placed in a 2 mL microtube (Greiner Bio-One, Germany; RNase—and DNase-free) and stored at −70 °C. These samples were later used to quantitatively assess antioxidant enzyme activity and apoptotic markers using real-time PCR.

#### Biochemical analysis

Serum lipid profiles were analyzed using commercially available enzymatic colorimetric kits (Spectrum Diagnostics, Egypt) according to the manufacturer’s instructions. The following parameters were measured: Total Cholesterol: Determined using a colorimetric assay kit according to Tietz and Berger^59^ (Catalog No. 230 002, 4 × 25 mL). Triglycerides: Measured using a colorimetric assay kit according to McGowan et al.^60^ (Catalog No. 314 002, 4 × 25 mL). Low-Density Lipoprotein (LDL): Assessed using a colorimetric assay kit according to Young^61^ (Catalog No. 280 002, 200 tests). High-Density Lipoprotein (HDL): Determined using a colorimetric assay kit according to Young^61^ (Catalog No. 267 002, 200 tests). All analyses were conducted according to the manufacturer’s protocols.

The concentration of malondialdehyde (MDA) in the ovarian homogenate was determined using a colorimetric method with a kit from Biodiagnostic Company, Egypt (Catalog No. MD 25 29). Nitric oxide (NO) content in the ovarian homogenate was assessed using a colorimetric method and a kit from Biodiagnostic Company, Egypt (Catalog No. NO 25 33).

Catalase (CAT) activity in the ovarian homogenate was measured using a colorimetric approach with a CAT kit from Biodiagnostic Company, Egypt (Catalog No. CA 25 17). Superoxide dismutase (SOD) activity in the ovarian homogenate was also measured using a colorimetric method and an SOD kit from Biodiagnostic Company, Egypt (Catalog No. SD 25 21).

The levels of TNF-α in plasma were quantified using the sandwich enzyme immunoassay technique, following the manufacturer’s instructions for the RayBiotech TNF-α ELISA kit (Catalog No. ELM-TNF-α, RayBiotech, Norcross, GA, USA). The amount of TNF-α in the sample was directly proportional to the color intensity measured at 450 nm, and results were expressed in pg/mL.IL-6 levels in serum were measured with a mouse ELISA kit (Catalog No. ELM-IL1b and ELM-IL6, RayBiotech, Norcross, GA, USA). A mouse IL-6-specific antibody was used following the kit’s instructions. IL-6 levels were proportional to the color intensity at 450 nm and expressed in pg/ml.

LH in rat serum was evaluated using Cobas ELISA kits (Catalog No. 11732234122, Cambridge, MA, USA). LH levels were reported in mIU/mL. Testosterone levels in rat serum were assessed using the Cobas Mouse/Rat Testosterone ELISA Kit (Catalog No. 05200067 190 100, Cambridge, MA, USA), with serum testosterone levels reported in pg/mL.

Anti-Müllerian hormone (AMH) in rat serum was quantitatively determined using Cobas ELISA kits (Catalog No. 07957190 190 100, Cambridge, MA, USA), with results reported in ng/mL.

In rat serum, follicle-stimulating hormone (FSH) was measured using Cobas ELISA kits (Catalog No. 07027346190 300, Cambridge, MA, USA), with FSH levels expressed in mIU/mL.

Estradiol (E2) levels in rat serum were determined using an ELISA kit (Catalog No. MBS2509833, Cambridge, MA, USA), with results reported in mIU/mL.

Insulin hormone levels in serum were estimated using a kit from Cobas Company (Catalog No. 12017547 122 100, Cambridge, MA, USA), and insulin concentrations were expressed in µU/mL.

Serum insulin-like growth factor-1 (IGF-1) levels were measured using a kit from Cobas Company (Catalog No. 07475896 190 100, Cambridge, MA, USA).

For flow cytometry analysis of apoptosis and cell cycle regulation, cells were fixed and permeabilized, incubated with mouse anti-P53 "aa20-25" FITC (Clone: DO-1) for P53 detection and FITC rabbit anti-active caspase-3 (CPP32; Yama; Apopain, BD Biosciences) to analyze caspase-3. Cells were washed with PBS/BSA, centrifuged at 400 × g, resuspended in 0.5% paraformaldehyde, and analyzed via flow cytometry.

Total RNA was extracted from ovarian tissue using the High Pure RNA Isolation Kit (Cat. No. 11 828 665 001, Roche Diagnostics GmbH, Germany) according to the manufacturer’s protocol for subsequent quantitative real-time PCR (q RT PCR) analysis of the Amh2 gene. Primer sequences for the genes analyzed are detailed in Table 4.

**Table. 4:** Primer sequence for genes.

Gene	Primer sequences
AMHR2	F: GGGAGCGTTGGCAGGAT R: CACATGACCTATCTTCCCGAATG Rat AMHR2 qPCR Primer Pair Cat: RP300065
β-Actin	F: CCGTGAAAAGATGACCCAGATC Rat beta-Actin qPCR Primer Pair R: CACAGCCTGGATGGCTACGT Cat: RP301046

### Histopathological examinations

After the experiment, four rats per group were anesthetized, euthanized, and dissected. The uterus and left ovaries were fixed in Bouin’s solution for one week, cleared in xylene, dehydrated in graded ethanol, and embedded in paraffin wax at 58–60 °C. Sections (5 µm) were stained with hematoxylin and eosin for histopathological examination.

### Immunohistochemical analysis for Ki-67 and PCNA

Ovarian tissue sections underwent immunohistochemistry processing. Dewaxed sections were hydrated and incubated in pH 8 EDTA antigen retrieval solution, followed by 0.3% hydrogen peroxide to inhibit endogenous peroxidase activity and protein blocking. Sections were then processed with primary antibodies against PCNA (Catalog No. PA5-27,214, 1:300 dilution) and Ki-67 (R&D Systems Inc., Minneapolis, MN, USA; 1:100 dilution).

After three PBS washes, sections were treated with anti-mouse IgG secondary antibody (EnVision + System HRP; Dako) for 30 min at room temperature. Immunoreactivity was visualized using the DAB substrate system (Liquid DAB + Chromogen; Dako) and counterstained with Mayer’s hematoxylin. Normal mouse serum replaced primary antibodies as a negative control. Stained sections were analyzed and photographed using an Olympus® microscope to evaluate immunohistochemical staining and morphological changes.

### Reproductive performance

Four female rats per group were paired with fertile Sprague Dawley male rats (2 males: 4 females) for two months. Successful mating was confirmed by a vaginal copulatory plug or spermatozoa in vaginal smears. Pregnant females were housed individually until delivery. Rats failing to conceive or give birth within two months were deemed infertile, according to Ozer et al.^62^.

The fertility success percentage was estimated using the following formula: fertility success% = number of impregnated females / total number of mated females × 100^63^. In addition, the duration of maternity was estimated by subtracting the gestation period (typically 21 days) from the interval between the day of mating and the day of delivery (A): (Maternity period = A-21)^62^. Pups were counted and weighed at birth, and their crown rump length (the straight distance from the uppermost point of the head to the base of the tail) was measured and statistically quantified. Healthy status and any visible abnormalities were also determined.

### Statistical analysis

All statistical analyses were performed using GraphPad Prism 8.0. The findings are displayed as the percentage of change and the mean ± standard error of the mean (SEM). One-way analysis of variance (ANOVA) was used for statistical comparisons, and Tukey was used as a post-hoc test.

No trend analysis was performed, as the study was not designed as a longitudinal or dose–response investigation.

## Data Availability

The datasets generated and/or analyzed during the current study are available from the corresponding author upon reasonable request.
